# Down-sampling in diffusion MRI: a bundle-specific DTI and NODDI study

**DOI:** 10.3389/fnimg.2024.1359589

**Published:** 2024-03-28

**Authors:** Federico Spagnolo, Susanna Gobbi, Enikő Zsoldos, Manon Edde, Matthias Weigel, Cristina Granziera, Maxime Descoteaux, Muhamed Barakovic, Stefano Magon

**Affiliations:** ^1^Roche Pharma Research and Early Development, Neuroscience and Rare Diseases, Roche Innovation Center, Basel, Switzerland; ^2^Sherbrooke Connectivity Imaging Lab (SCIL), Université de Sherbrooke, Sherbrooke, QC, Canada; ^3^Imeka Solutions Inc, Sherbrooke, QC, Canada; ^4^Translational Imaging in Neurology (ThINK) Basel, Department of Biomedical Engineering, Faculty of Medicine, University Hospital Basel and University of Basel, Basel, Switzerland; ^5^Department of Neurology, University Hospital Basel, Basel, Switzerland; ^6^Research Center for Clinical Neuroimmunology and Neuroscience Basel (RC2NB), University Hospital Basel and University of Basel, Basel, Switzerland; ^7^Division of Radiological Physics, Department of Radiology, University Hospital Basel, Basel, Switzerland

**Keywords:** MRI, neurodegenerative diseases, acquisition time, DTI, NODDI

## Abstract

**Introduction:**

Multi-shell diffusion Magnetic Resonance Imaging (dMRI) data has been widely used to characterise white matter microstructure in several neurodegenerative diseases. The lack of standardised dMRI protocols often implies the acquisition of redundant measurements, resulting in prolonged acquisition times. In this study, we investigate the impact of the number of gradient directions on Diffusion Tensor Imaging (DTI) and on Neurite Orientation Dispersion and Density Imaging (NODDI) metrics.

**Methods:**

Data from 124 healthy controls collected in three different longitudinal studies were included. Using an in-house algorithm, we reduced the number of gradient directions in each data shell. We estimated DTI and NODDI measures on six white matter bundles clinically relevant for neurodegenerative diseases.

**Results:**

Fractional Anisotropy (FA) measures on bundles where data were sampled at the 30% rate, showed a median *L*_1_ distance of up to 3.92% and a 95% CI of (1.74, 8.97)% when compared to those obtained at reference sampling. Mean Diffusivity (MD) reached up to 4.31% and a 95% CI of (1.60, 16.98)% on the same premises. At a sampling rate of 50%, we obtained a median of 3.90% and a 95% CI of (1.99, 16.65)% in FA, and 5.49% with a 95% CI of (2.14, 21.68)% in MD. The Intra-Cellular volume fraction (ICvf) median *L*_1_ distance was up to 2.83% with a 95% CI of (1.98, 4.82)% at a 30% sampling rate and 3.95% with a 95% CI of (2.39, 7.81)% at a 50% sampling rate. The volume difference of the reconstructed white matter at reference and 50% sampling reached a maximum of (2.09 ± 0.81)%.

**Discussion:**

In conclusion, DTI and NODDI measures reported at reference sampling were comparable to those obtained when the number of dMRI volumes was reduced by up to 30%. Close to reference DTI and NODDI metrics were estimated with a significant reduction in acquisition time using three shells, respectively with: 4 directions at a b value of 700 *s*/*mm*^2^, 14 at 1000 *s*/*mm*^2^, and 32 at 2000 *s*/*mm*^2^. The study revealed aspects that can be important for large-scale clinical studies on bundle-specific diffusion MRI.

## 1 Introduction

Diffusion MRI (dMRI) is used for studying white matter (WM) alterations by extracting parameters that are associated with tissue microstructure (e.g., intra-cellular axonal volume fraction). Different approaches were developed to model the diffusion process of water molecules in tissue, either using single models, i.e., diffusion tensor imaging (DTI) (Basser et al., 1994a,b), or multiple-compartment models such as the Neurite Orientation Dispersion and Density Imaging (NODDI) (Zhang et al., 2012; Winston, 2015) and the Composite Hindered and Restricted Model of Diffusion (CHARMED) (Assaf and Basser, 2005).

DTI measures can quantify the diffusion coefficient of water molecules in different directions inside the brain. This information is sensitive to properties of tissue microstructure, such as the level of myelination, axon, and cell density (Schilling et al., 2019). DTI metrics estimate different quantities and show various tissue physical properties. Namely, the mean diffusivity (MD) or apparent diffusion coefficient (ADC) evaluates the overall water diffusion at the voxel-wise level, by averaging the elements along the tensor diagonal. Fractional anisotropy (FA) measures the degree of diffusion asymmetry, while radial diffusivity (RD) reflects the diffusivity perpendicular to axonal fibers, i.e., the average of the smaller elements on the tensor diagonal.

Still, the DTI model presents some sensitivity and specificity limitations (Tournier et al., 2011; Minosse et al., 2021) that can be overcome using multi-compartment models, which help describe more complex WM fiber orientations than a diffusion tensor. With these types of models it is possible to increase the angular resolution of the orientational information and describe crossing fibers within a voxel. In these cases, diffusion-encoding images can be represented as sampling points on a spherical shell (Aganj et al., 2015).

One of the most popular multi-compartment models is NODDI. It divides brain tissue into three compartments: intra-cellular, extra-cellular, and CSF. Each one represents different characteristics and reflects a unique behavior of water diffusion. The intra-cellular compartment models the space bounded by membranes of neurites as a set of cylinders of zero radii, reflecting unhindered diffusion along neurites and the highly restricted diffusion in the orthogonal direction. The extra-cellular compartment represents the space outside of the membrane of neurites, which is characterized by the diffusion of water molecules hindered by neurites, and therefore is modeled as a Gaussian anisotropic diffusion process. The CSF compartment refers to the cerebrospinal fluid space and is described by isotropic Gaussian diffusion using a constant diffusivity parameter.

To analyze white matter bundles, further steps are necessary. The first step involves performing tractography, which is the reconstruction of fiber tracts. This process includes a computational procedure for determining the anatomical trajectories of the fiber tracts. There are several software solutions available with different fiber tracking algorithms; a comprehensive review of tractography and its approaches can be found in a recent publication (Zhang et al., 2022). The second step is tractometry, which involves estimating the distribution of microstructural measurements along fiber tracks (Yeatman et al., 2012). Recent research, such as Edde et al. (2023), has evaluated the variability and reproducibility of DTI and NODDI metrics in white matter bundles.

However, these approaches present some underlying critical inefficiencies that often jeopardize the use of MRI in clinical research. Namely, the lengthy acquisition time and the increased risk of movement artifacts during long protocols (Hollingsworth, 2015).

Attempts to reduce acquisition times have been made by, e.g., parallel imaging (Feinberg and Setsompop, 2013), where down-sampling takes place in k-space, or using post-processing reconstruction methods (Lustig et al., 2007). Another study (Caruyer et al., 2013) developed a sampling method to cover spherical shells via non-convex optimisation, i.e., treating gradient directions as electrical charges and selecting the distribution to minimize the electrostatic repulsion. Another possible way is reconstructing the signal from a much smaller number of measurements, e.g., through compressive sampling (Michailovich and Rathi, 2010). The effects of different sampling schemes have been reported on the computation of orientational-average (Afzali et al., 2021a) and the sensitivity to microstructural properties (Afzali et al., 2021b).

This study investigated the possibility of saving acquisition time in dMRI protocols by concomitantly reducing the number of acquired directions and estimating metrics comparable to those obtained at reference sampling. To do so, we assessed the influence of the number of gradient directions on standard DTI (Mean Diffusivity [MD] and Fractional Anisotropy [FA]) and NODDI measures (Intra-Cellular volume fraction [ICvf]) in three data sets with different multi-shell diffusion protocols.

## 2 Materials and methods

### 2.1 Subjects and MRI protocol

We analyzed MRI data from one public source (adni.loni.usc.edu) and two data sets collected in collaboration with the University of Sherbrooke (Canada) and the University of Basel (Switzerland), respectively. Written consent was obtained from all subjects participating in the studies according to the Declaration of Helsinki (WMA, 2001), and the study was approved by the institutional review board at each participating site.

Throughout this and the following sections, the term “reference sequence” means the originally acquired number of gradient directions, while down-sampled data will be indicated by specifying the respective sampling rate.

Data set 1 consisted of a subset of 93 healthy subjects collected in ADNI3^1^ (mean age 75.64 years ± 8.42, 64 females) with three annual visits. Data were collected using three 3T Siemens scanners (Prisma, Prisma Fit, and Skyra). The dMRI reference sequence included three *b* values and lasted for 7 min and 20 s (Table 1).

**Table 1:** Description of reference sequences.

	**ADNI3**	**Sherbrooke**	**Basel**
*b* = 0 *s*/*mm*^2^	13	7	12
*b* = 300 *s*/*mm*^2^	–	8	–
*b* = 500 *s*/*mm*^2^	6	–	–
*b* = 700 *s*/*mm*^2^	–	–	6
*b* = 1,000 *s*/*mm*^2^	48	32	20
*b* = 2,000 *s*/*mm*^2^	60	60	45
*b* = 3,000 *s*/*mm*^2^	–	–	66
δ (ms)	13.6	21.9	19
Δ (ms)	35	46.5	36
Voxel resolution (mm)	2 × 2 × 2	2 × 2 × 2	1.8 × 1.8 × 1.8
TR (ms)	3,300	4,800	4,500
TE (ms)	71	92	75
Field strength (T)	3	3	3
Acquisition type	2D	2D	2D
Total time	7 min 20 s	9 min 19 s	15 min 18 s

Each column represents a different data set, while rows show the acquisition parameters, such as *b* values, small and big deltas, the voxel resolution, repetition and echo time, and the estimated acquisition time.

Data set 2 included 19 healthy controls (HC) (mean age 36.15 years ± 4.8, 19 females), with five visits per participant acquired over five months at the University of Sherbrooke. The diffusion protocol contained three shells of data obtained in 9 min and 19 s using a Philips 3T Ingenia scanner (Table 2) (Edde et al. (2022)).

**Table 2:** List of sampled gradient directions.

	**ADNI3**	**Sherbrooke**	**Basel**
**Reference**	**6–48–60**	**8–32–60**	**6–20–45**
10%	5–43–54	7–29–54	5–18–41
30%	4–34–42	6–22-42	4–14–32
50%	3–24–30	4–16–30	3–10–23

Each column represents a different data set, while rows refer to sampling protocols, starting from the reference sequence to 10%, 30% and 50% down-samplings. Each element of the table shows the number of directions in the first, second, and third shells. The *b*0 (having *b* = 0 s/mm^2^) shells are not included as they were not down-sampled.

Data set 3 consisted of 12 healthy subjects (mean age 31 years ± 8.72, 6 females), with three visits for each subject acquired at the University of Basel. Two sessions were collected on the same day without repositioning the subject, and a third session was collected one week apart. The diffusion protocol included four shells of data acquired in 15 min and 18 s using a Siemens 3T Prisma scanner (Table 1) (Rahmanzadeh et al. (2022)).

### 2.2 Down-sampling of gradient directions

To identify the minimal number of gradient directions that allowed us to compute DTI and NODDI metrics with down-sampled data, an algorithm (Caruyer et al., 2013) that uniformly removed directions across *b* values was adopted.

Briefly, the algorithm used a generalization of the electrostatic repulsion to optimize the angular distribution of gradient directions. Their cost function


(1) $$\begin{array}{l} V=\alpha V_{1}+\left(1-\alpha \right)V_{2} \end{array}$$

contained two sub-functions to be minimized through a non-convex optimisation problem. In Equation 1, the first term *V*_1_ acted on directions in each separate shell, while *V*_2_ referred to the whole set of directions (i.e., once all directions are projected onto a single unit sphere). The optimisation forced, in both cases, a uniform distribution of gradient directions. This choice is based on literature findings (Zhan et al., 2011; Koay et al., 2012; Ye et al., 2012), recommending to obtain sampling points as different as possible from one shell to another, for an improved angular resolution.

Ultimately the sub-functions depended on the number of shells, the number of directions per shell, and the directions' coordinates. The parameter α identified a weight to balance the importance of global and per-shell coverage, and it was set to 0.5. The code was implemented in scilpy (https://github.com/scilus/scilpy).

**Figure 1:**
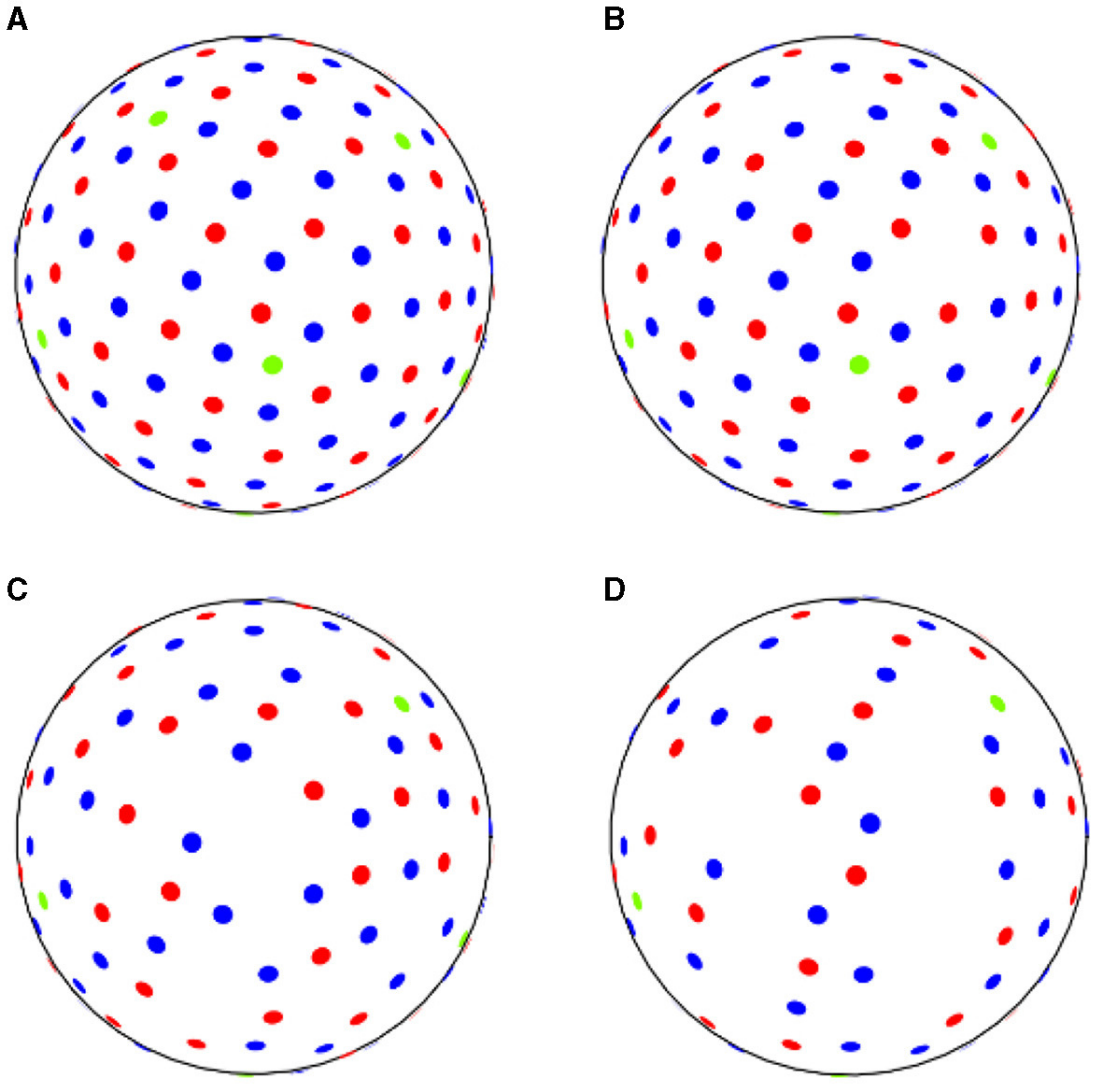
ADNI3 data samples acquired **(A)** with reference sequence, **(B)** down-sampling at 10% rate, **(C)** at 30% rate, **(D)** at 50% rate. Green dots correspond to *b*-values of 500 *s*/*mm*^2^, red dots to *b*-values of 1,000 *s*/*mm*^2^ and blue dots to *b*-values of 2,000 *s*/*mm*^2^.

We set 10%, 30%, and 50% for the down-sampling thresholds. As an example, in the ADNI3 protocol, from a reference set of three shells with 6–48–60 directions, we sampled respectively 5–43–54 (10%), 4–34–42 (30%), and 3–24–30 (50%) points, as shown in Table 2 and Figure 1.

### 2.3 Pre-processing and bundles' reconstruction

Both the reference and the down-sampled data were run through the same processing pipeline consisting of:

1. Denoising with *dwidenoise*,
2. Field inhomogeneity and eddy currents correction with *dwifslpreproc* (Andersson et al., 2003; Smith et al., 2004; Andersson and Sotiropoulos, 2015),
3. Bias field correction with *dwibiascorrect ants* (Avants et al., 2011).

All these steps were performed using MRTrix 3.0 software package (Tournier et al., 2019). Additionally, brain masks were extracted with (HD-BET) (Isensee et al., 2019). The left/right orientation of all the images was ensured to be the same as that of the standard Montreal Neurological Institute (MNI) space (Evans et al., 2012), applying a rigid registration.

Fiber tracts segmentation was performed using the TractSeg tool (Wasserthal et al., 2018a,b, 2019, 2020), to generate streamlines of 40 WM bundles. For this study, we averaged metrics from the left and right parts of the same bundles; the Corpus Callosum was divided into five distinct parts (Anterior body, Posterior body, Rostrum and Genu, Splenium, and Isthmus), obtaining a total of 22 distinct bundles.

Furthermore, we evaluated anatomical measures on tracts (tractometry) through the following steps (Chandio et al., 2020):

1. Resampling of all streamlines to an equal number, i.e., 98, of segments/points.
2. Centroid estimation for all streamlines.
3. Assignment of each segment to the closest centroid segment.
4. Evaluation of a considered metric at each segment.
5. For each centroid segment, averaging the metric for all assigned streamline segments.

**Figure 2:**
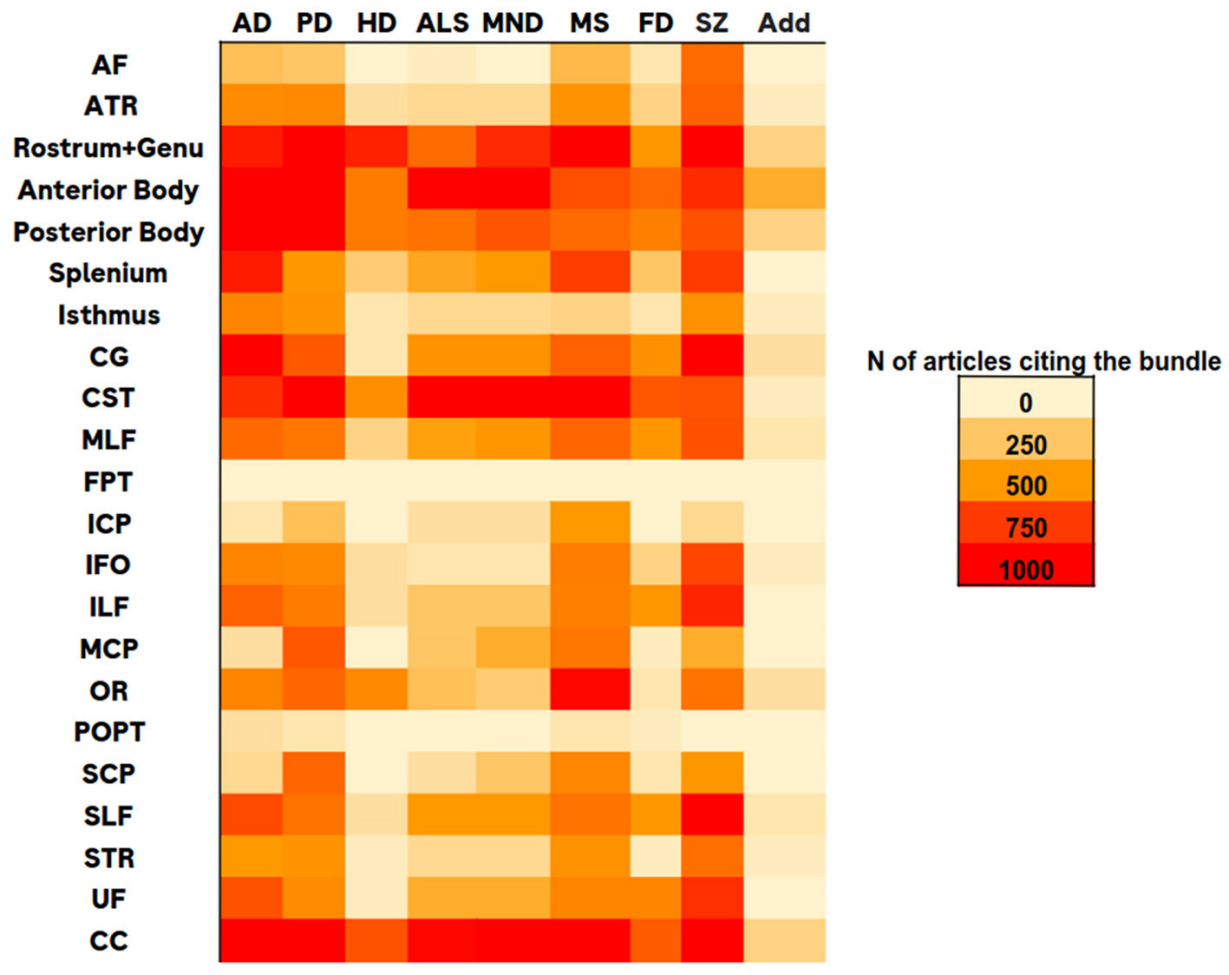
Heatmap table representing number of citations of bundles (rows) relatively to various neurodegenerative diseases (columns), where: AD, Alzheimer disease; PD, Parkinson disease; HD, Huntington disease; ALS, amyotrophic lateral sclerosis; MND, motor neuron disease; MS, multiple sclerosis; FD, frontotemporal dementia; SZ, schizofrenia and Add for addictive disorders.

Using the library PyMed (https://github.com/gijswobben/pymed), we accessed publications in PubMed (https://pubmed.ncbi.nlm.nih.gov/) and screened the number of citations for a specific bundle and a neurodegenerative disease (NDD). As shown in Figure 2, we found the following bundles as the most relevant for NDDs: Corticospinal Tract (CST), Inferior Fronto-Occipital fasciculus (IFO), Inferior Longitudinal Fasciculus (ILF), Optic Radiation (OR), Superior Longitudinal Fasciculi (SLFI, SLFII, SLFIII) and Corpus Callosum (CC), composed by Splenium, Rostrum, and Genu (RG), Isthmus, Anterior and Posterior Body.

### 2.4 Bundle average value

For each bundle, several diffusion metrics were assessed. We generated maps for mean diffusivity (MD), fractional anisotropy (FA), and Radial diffusivity (RD). These maps were generated with MRTrix 3.0, by estimating the diffusion tensor using the *dwi2tensor* function (https://mrtrix.readthedocs.io/en/dev/reference/commands/dwi2tensor.html). Furthermore, we evaluated indices of microstructural changes in brain tissues described in the NODDI model, using the Accelerated Microstructure Imaging via Convex Optimization (AMICO) framework (https://github.com/daducci/AMICO/wiki) (Daducci et al., 2015): the intra-cellular volume fraction (ICvf), the isotropic volume fraction (ISOvf), and the orientation dispersion (OD) (Zhang et al., 2012; Winston, 2015).

For all metrics, we estimated one value for each bundle (averaging across segments) and calculated the *L*_1_ distance, i.e., the absolute value of the difference, between the value obtained from the reference and each down-sampled sequence. Then, each distance was divided by the reference value to obtain a relative difference, and expressed as a percentage. To compare values in the three data sets, we plotted their distribution across sessions, separately for each bundle.

### 2.5 Bundle profile

Averaging metric values across segments of a bundle did not take into account how these values were distributed along the tract (profile). Thus, a bundle profile was reported by plotting the values in each segment of the metric, at all sampling rates.

The distribution of bundles profiles (and average bundles value) across subjects was evaluated with a two-sample Kolmogorov–Smirnov (KS) test (α = 0.05) between the original and down-sampled protocol. We considered the metrics value on the same bundle segment across all subjects. The resulting *p*-values were corrected for false discoveries rate (FDR threshold 0.05).

### 2.6 Agreement and variability of sampling methods

We investigated the agreement, i.e., the degree of concordance between sets of measurements, and the within-subject variability using the Bland–Altman plot (Bland and Altman, 1986, 1995, 1999).

This plot shows a solid line (estimated bias) corresponding to the average difference between the two methods. Two dashed lines represent the average difference ± 1.96 standard deviations (SD), also called limits of agreement (LoA), and estimate the variability of the differences. The authors' recommendation is that 95% of difference data should lie in between these two limits so that the difference between the two methods is normally distributed (Gaussian) (Giavarina, 2015). Furthermore, a basic assumption of the method is that all measurements must be independent.

Agreement and variability analyses were performed on the average FA, MD, and ICvf values obtained on CC and CST in all subjects and visits. Specifically, we designated values from reference data as method A and values from each down-sampled data as method B.

Within-subjects variability was performed using MedCalc for Windows, version 20.112 (MedCalc Software, Ostend, Belgium; https://www.medcalc.org; 2022) on the two most studied bundles (CC and CST), reconstructed from Basel's data set. Plots of ADNI3's and Sherbrooke's subjects are reported in the Supplementary material.

### 2.7 Total volume of bundles

A quantitative analysis of WM volume between reference and down-sampled data was included by summing up the volume of all 22 segmented bundles. The volume of each bundle corresponded to the number of voxels in the bundle mask, which was reconstructed via TractSeg. We calculated the relative difference between the WM volume of reference and down-sampled data and derived the distribution across subjects and sessions (Macauley et al., 2015).

Results of the most relevant metrics are shown in the following section, including ICvf, FA, and MD. Additional metrics, i.e., ISOvf, OD, and RD, are included in the Supplementary material.

The impact of down-sampling on fiber tracts segmentation was evaluated with a two-sample KS test (α = 0.05) between the original and the down-sampled protocol. The resulting *p*-values were corrected for false discoveries.

## 3 Results

The comparison between metrics estimated in down-sampled and original protocols was reported here in four separate subsections. The first focused more generally on the average value of bundles; the second compared values in each segment of the bundles, i.e., their profiles; the third examined the level of variability and agreement of measurements; the fourth considered the union of bundles to evaluate the potential WM volume loss during bundles' reconstruction.

### 3.1 Bundles' average value

Here we reported the distribution of the *L*_1_ distance between the average values obtained from the reference and each down-sampled sequence for each bundle and metric. The results were expressed as a relative difference of the reference values.

**Figure 3:**
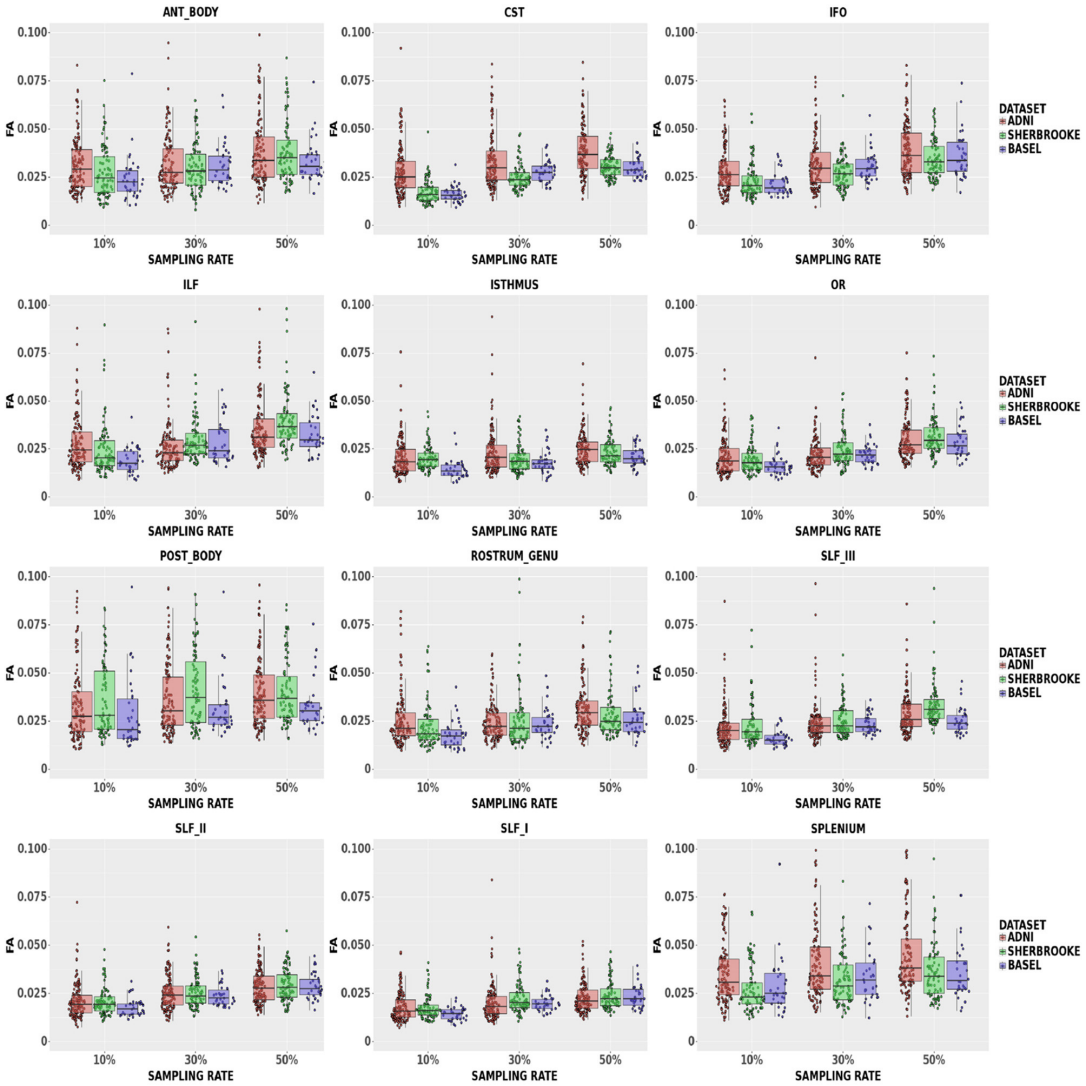
Average values in FA in different bundles comparing our three data sets. Colors of boxes represent different data sets: ADNI3 (red), Sherbrooke (green), Basel (blue). The *y*-axis refers to the subject distribution of the average values for FA in the following bundles: Anterior and Posterior Body, CST, IFO, ILF, Isthmus, OR, Rostrum and Genu, SLF, Splenium. The *x*-axis refers to the three adopted sampling rates.

FA: Figure 3 reported that down-sampling at 50% brought the median *L*_1_ distance up to 3.90% and a 95% CI of (1.99, 16.65)%. The bundles with lower *L*_1_ distance were SLF, OR, Isthmus, and RG, with a median ranging from a maximum of 2.11% and a 95% CI of (1.15, 6.79)% at 10% sampling to 3.15% and a 95% CI of (2.30, 6.03)% at 50% sampling. The 95% CI was wider in the Splenium, Anterior, and Posterior Body. Respectively, these bundles reported values of (1.99, 16.65)%, (1.63, 9.56)%, and (1.80, 12,01)% in ADNI3 data set at 50% sampling.

**Figure 4:**
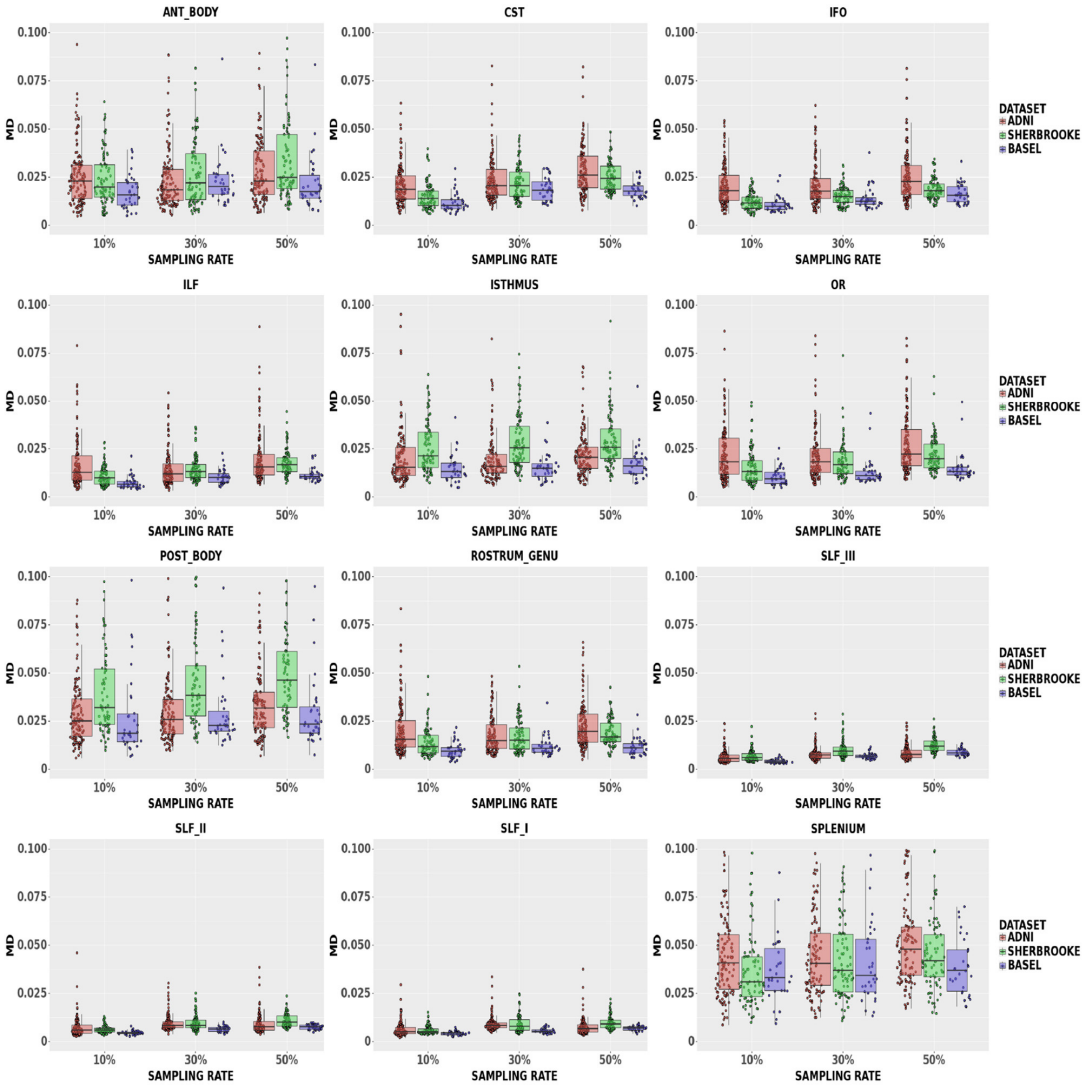
Average values in MD in different bundles comparing our three data sets. Colors of boxes represent different data sets: ADNI3 (red), Sherbrooke (green), Basel (blue). The *y*-axis refers to the subject distribution of the average values for MD in the following bundles: Anterior and Posterior Body, CST, IFO, ILF, Isthmus, OR, Rostrum and Genu, SLF, Splenium. The *x*-axis refers to the three adopted sampling rates.

MD: Figure 4 showed that, at a 50% sampling rate, Sherbrooke data provided a median of 5.49% with a 95% CI of (2.14, 21.68)% in the Posterior Body, and ADNI3 performed similarly in the Splenium with 5.18% and a 95% CI of (2.03%, 17.42)%. The median in other bundles reached around 2.61% with a 95% CI of (1.08, 19.69)% in the worst case; in SLF, the median at any sampling rate did not go beyond 1.22%. The 95% CI of some CC sections (Splenium, Anterior, and Posterior Body) was significantly higher, such as (0.71, 7.26)%, compared to other bundles.

**Figure 5:**
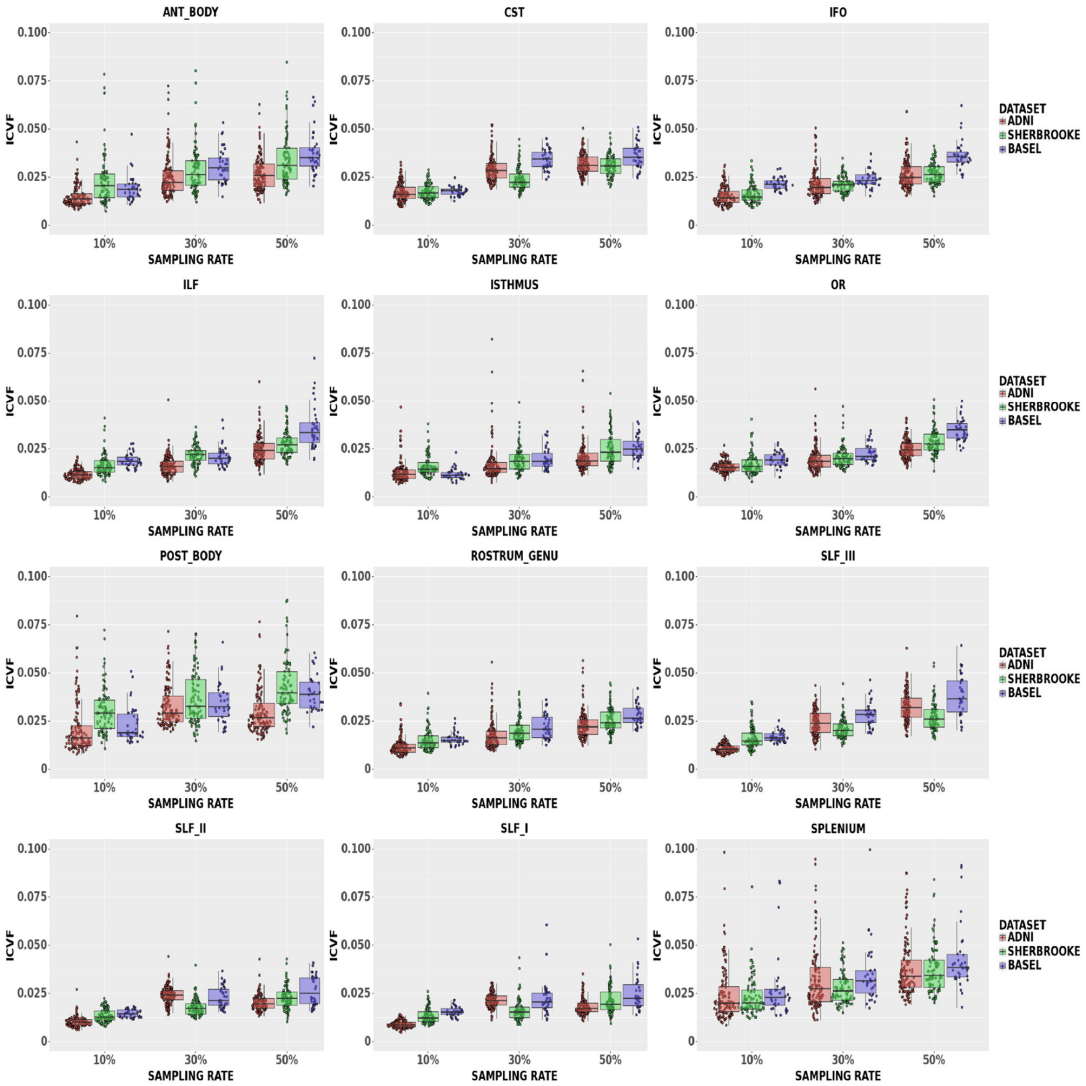
Average values in ICvf in different bundles comparing our three data sets. Colors of boxes represent different data sets: ADNI3 (red), Sherbrooke (green), Basel (blue). The *y*-axis refers to the subject distribution of the average values for ICvf in the following bundles: Anterior and Posterior Body, CST, IFO, ILF, Isthmus, OR, Rostrum and Genu, SLF, Splenium. The *x*-axis refers to the three adopted sampling rates.

ICvf: findings in Figure 5 suggested that down-sampling up to 30% lead to a median up to 3.26% with a 95% CI of (1.75, 6.64)% in the Posterior Body, while up to 50% rate values reached up to 3.95% with a 95% CI of (2.39, 7.80)% in the same bundle. In the Isthmus, SLFI, SLFII, and RG, the median was lower and, at 50% rate, RG reached 2.44% with a 95% CI of (1.52, 4.00)%.

Statistical analysis of the average bundle values in all three data sets and metrics showed *p*-values > 0.05 for all bundles.

### 3.2 Bundles' profile

In the statistical analysis on all data sets, both MD and FA showed *p*-values > 0.05, with the only exception of segment 195 of OR down-sampled at 50% in Sherbrooke (please note that, since we joined left and right parts of a bundle together, the segments range from 1 to 196). In the case of ICvf in Basel, *p*-values were lower than the threshold on three segments of the Posterior Body and two segments of ILF at 10%. In Sherbrooke, the same was reported on one segment of RG, two of the Anterior and Posterior Body, five on OR, and eight on the Isthmus, all at 50% sampling. In ADNI3 significant *p*-values were found in several segments of bundles at 30 and 50%, especially Posterior Body, Isthmus, CST, and SLF.

**Figure 6:**
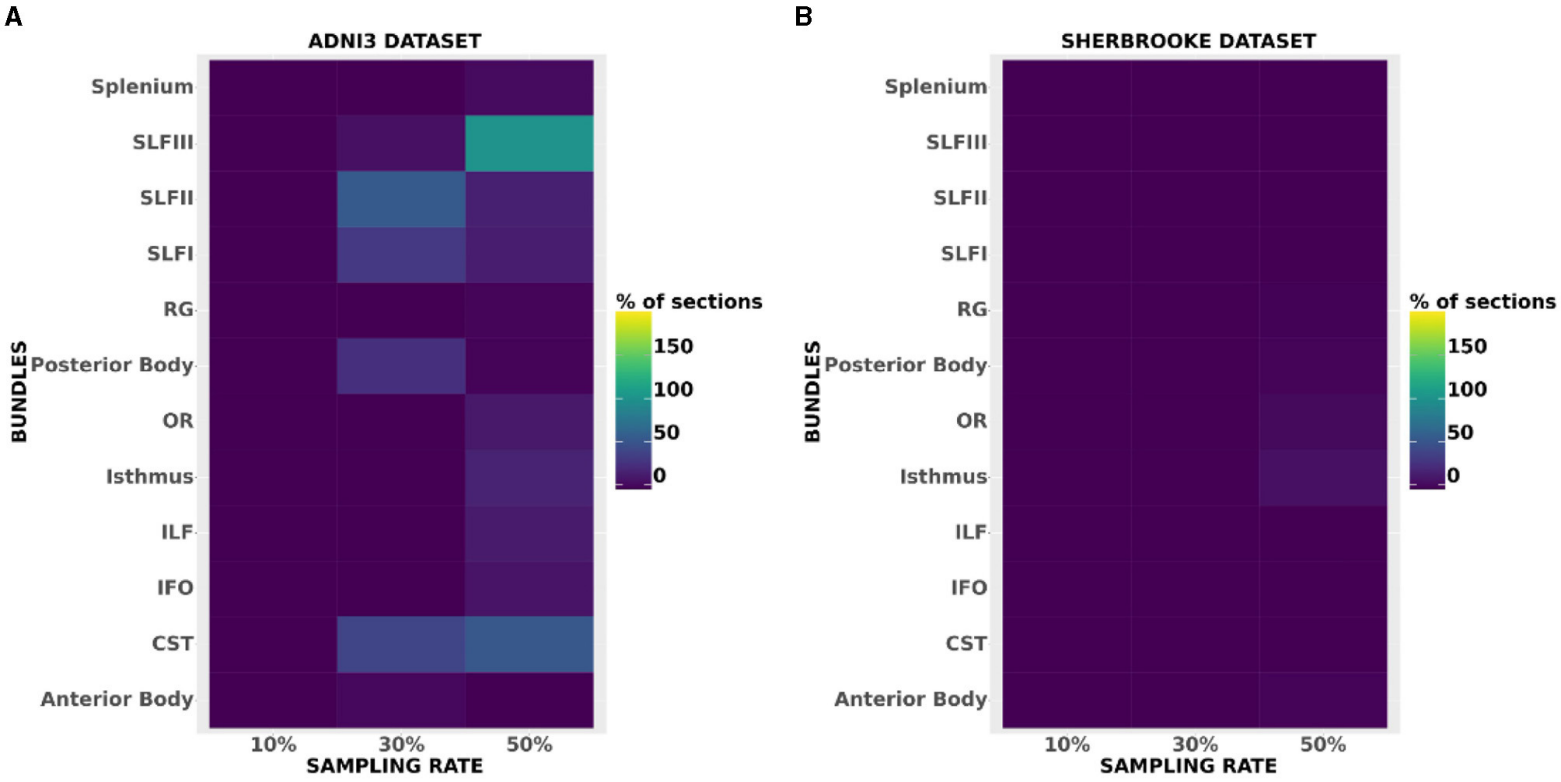
Heatmaps showing the amount of segments per bundle with significant *p*-values in the case of ICvf for ADNI3 **(A)** and Sherbrooke **(B)**. The *x*-axis reports different bundles while the *y*-axis differentiates the sampling rates.

**Figure 7:**
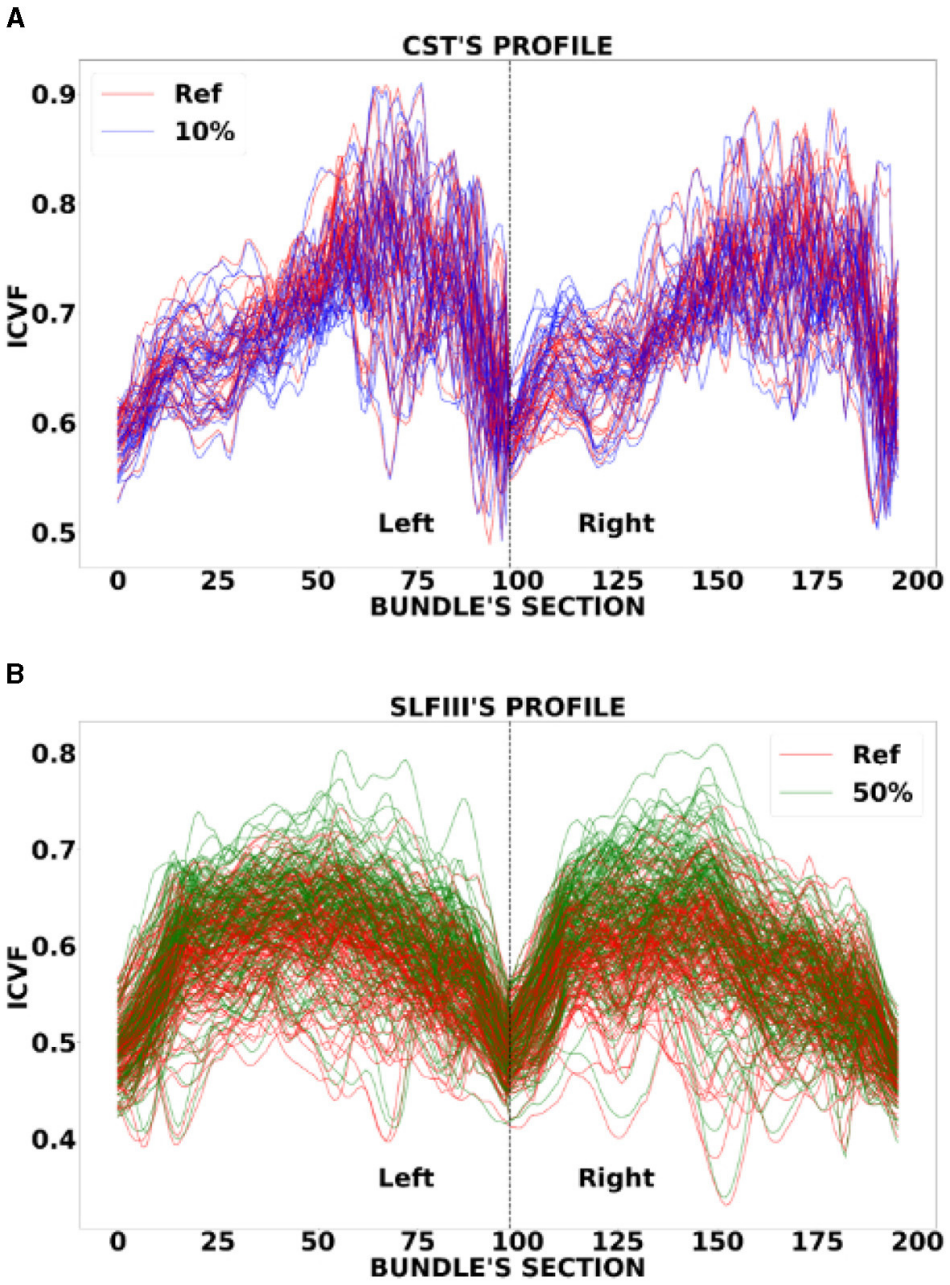
Examples of a bundle's ICvf profile reconstruction: **(A)** refers to CST in Basel at reference (red) and 10% sampling (blue), **(B)** refers to SLFIII in ADNI3 at reference (red) and 50% sampling (green). The *x*-axis represents the segments of the left and right bundles (196) and the *y*-axis represents the metric.

The heatmaps in Figure 6 compared the number of segments per bundle with significant *p*-values in the case of ICvf for ADNI3 and Sherbrooke, and at each sampling rate. Heatmaps obtained for other metrics and data sets did not report any visible difference from Figure 6. The distribution of ICvf across subjects in the Posterior Body (Basel data set) and in the SLFIII (ADNI3 data set) was illustrated in Figure 7.

### 3.3 Agreement and variability of sampling methods

**Figure 8:**
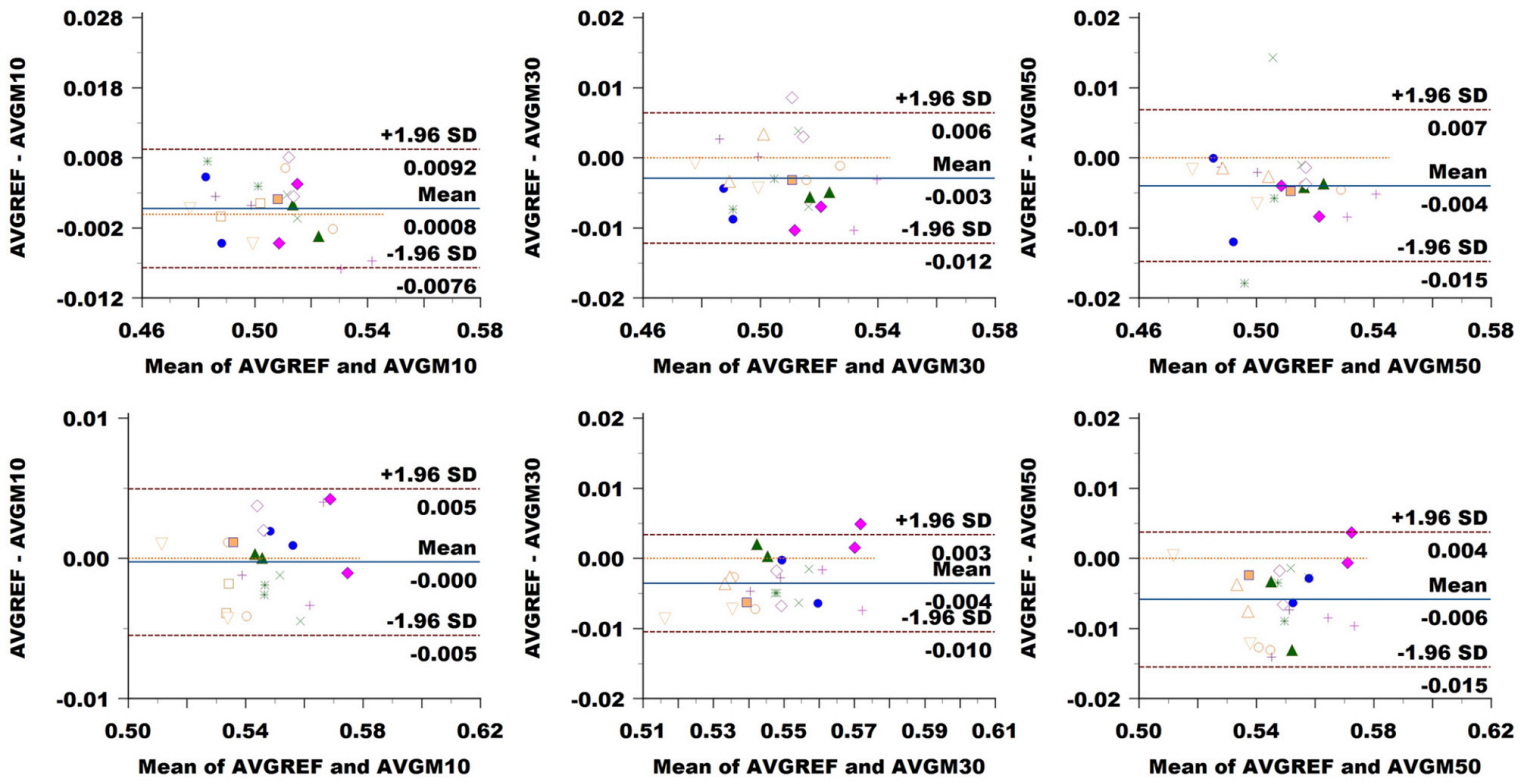
Bland–Altman plots of FA average values in CC (top row) and CST (bottom row) for Basel subjects. From left to right each plot in the columns compares data obtained from reference sequence (AVGREF) and data down-sampled at 10% (AVGM10), 30% (AVGM30) and 50% (AVGM50). The *x*-axis represents the mean of values measured by the models, the *y*-axis their difference. Visits of a same subject are plotted using the same marker. Blue solid lines correspond to the average difference while red dashed lines are the 95% limits of agreement.

FA: The Bland–Altman plot of the Basel data set (see Figure 8) revealed a bias in the CC (difference between methods) increasing with a down-sampling rate from 0.001 to –0.003 and –0.004. The limits of agreement (LoA) followed the same trend growing from ±0.008 to ±0.009 and up to 0.011 when halving the directions. CST showed no bias at 10% sampling, going up to –0.004 at 30% and –0.006 at 50%. LoA grew from ±0.005 to 0.007, reaching 0.010 at 50%.

**Figure 9:**
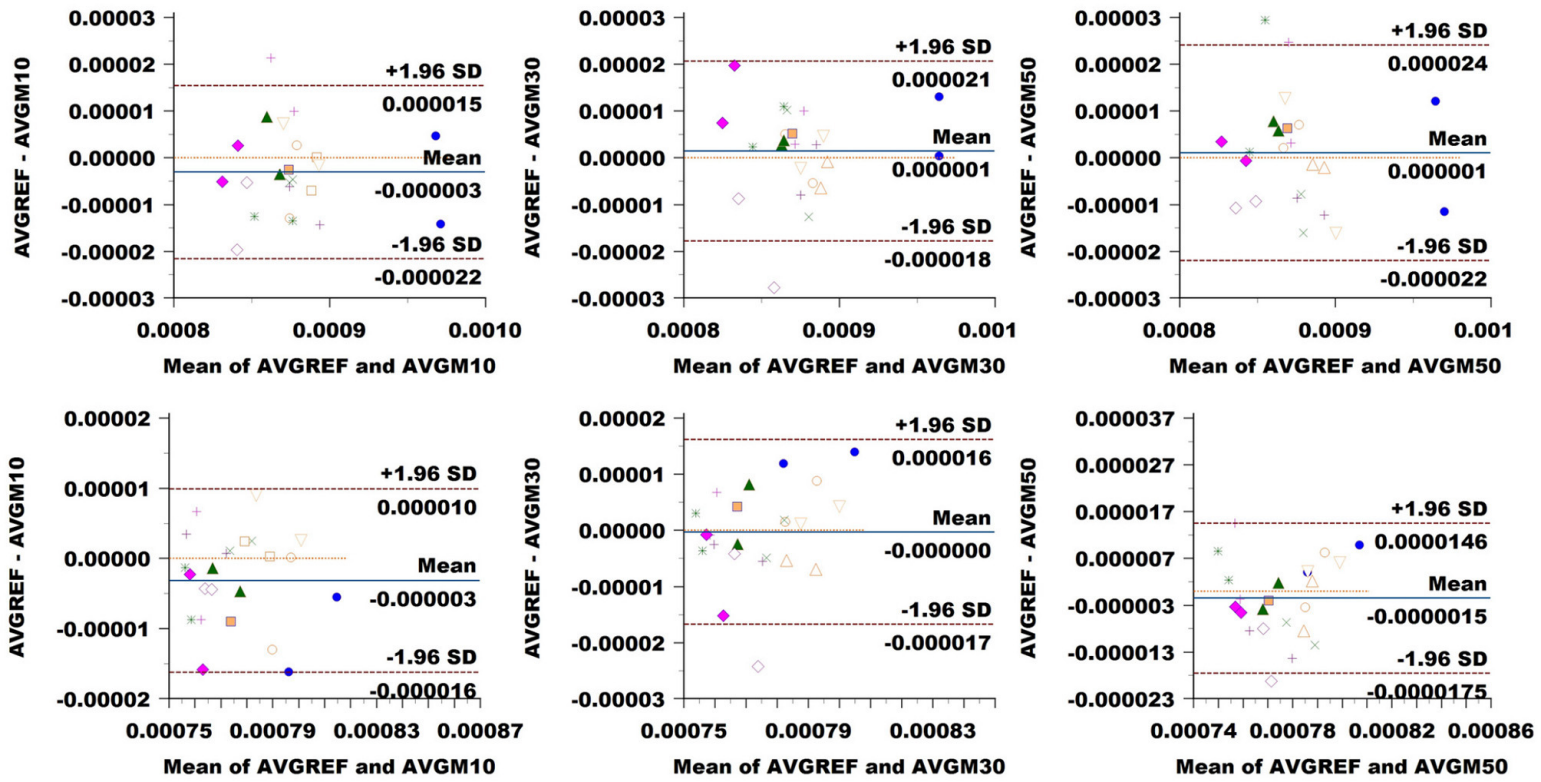
Bland–Altman plots of MD average values in CC (top row) and CST (bottom row) for Basel subjects. From left to right, each plot in the columns compares data obtained from the reference sequence (AVGREF) and data down-sampled at 10% (AVGM10), 30% (AVGM30) and 50% (AVGM50). The *x*-axis represents the mean values measured by the models, and the *y*-axis their difference. Visits of the same subject are plotted using the same marker. Blue solid lines correspond to the average difference, while red dashed lines are the 95% limits of agreement.

MD: Figure 9 showed a bias of ±3 × 10^−6^ in the CC when down-sampling at a 10% rate, compared to around ±10^−6^ at higher sampling rates. LoA exhibited growth in accordance with sampling rates from around 18 × 10^−6^ to 19 × 10^−6^ and 23 × 10^−6^. In CST, the bias seemed to be slightly higher at 10% sampling rate, while ±1.96 SD limits rose from 13 × 10^−6^ at 10% sampling to around 16 × 10^−6^ and 17 × 10^−6^ at higher rates.

**Figure 10:**
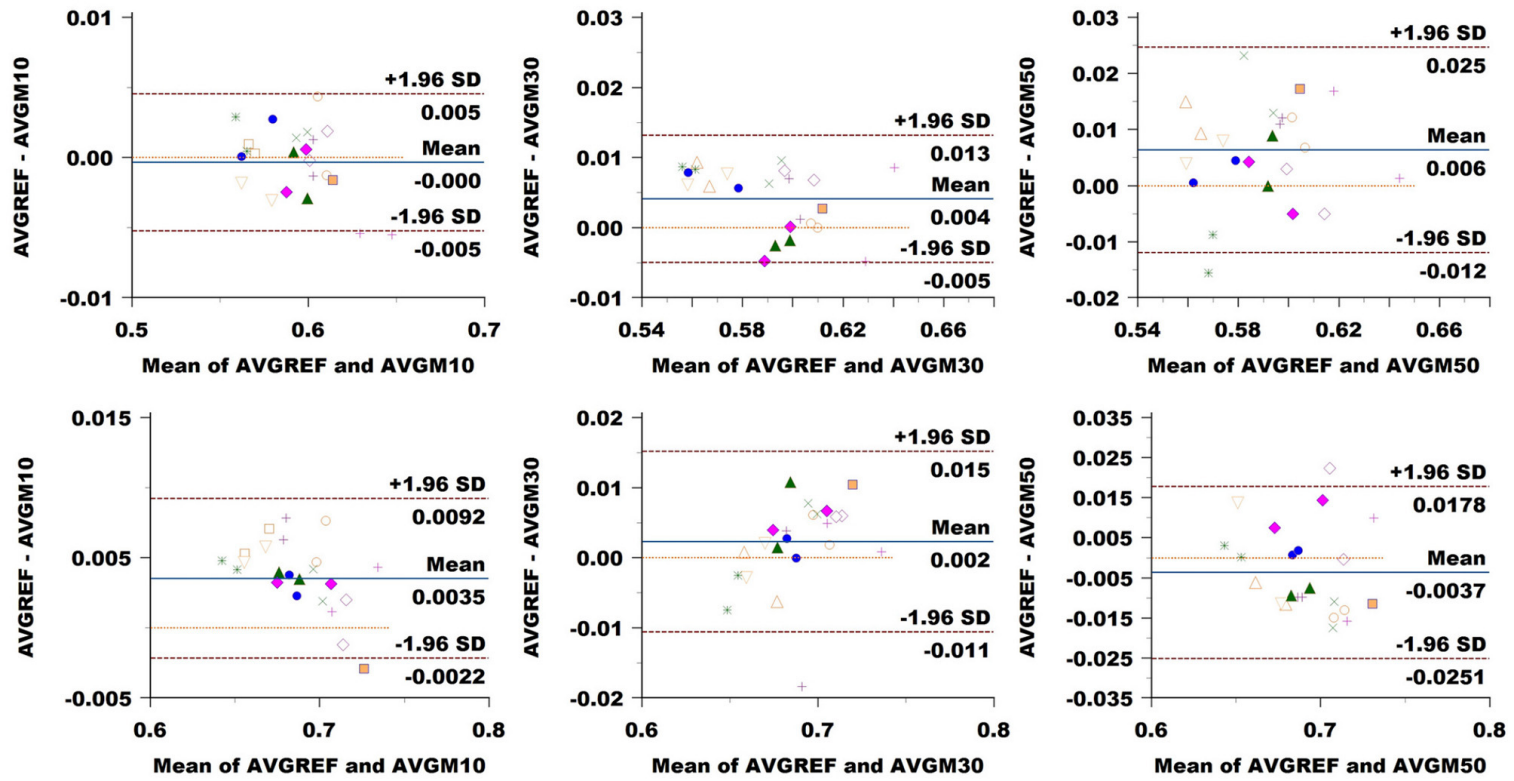
Bland–Altman plots of ICvf average values in CC (top row) and CST (bottom row) for Basel subjects. From left to right each plot in the columns compares data obtained from reference sequence (AVGREF) and data down-sampled at 10% (AVGM10), 30% (AVGM30) and 50% (AVGM50). The *x*-axis represents the mean of values measured by the models, the *y*-axis their difference. Visits of a same subject are plotted using the same marker. Blue solid lines correspond to the average difference while red dashed lines are the 95% limits of agreement.

ICvf: in CC, we observed no bias at a 10% sampling rate, while the comparison exhibited a value of 0.004 at 30% and 0.006 at 50% (Figure 10). LoA consistently widened, increasing the sampling rate from 0.005 at 10% to 0.009 at 30% and 0.021 at 50%. In CST, we noticed a bias of 0.004 at 10%, going slightly down at 0.002 at 30% and back up to –0.004 at 50%. LoA expanded with sampling rates from 0.005 to 0.013 and 0.022.

### 3.4 WM volume loss

**Figure 11:**
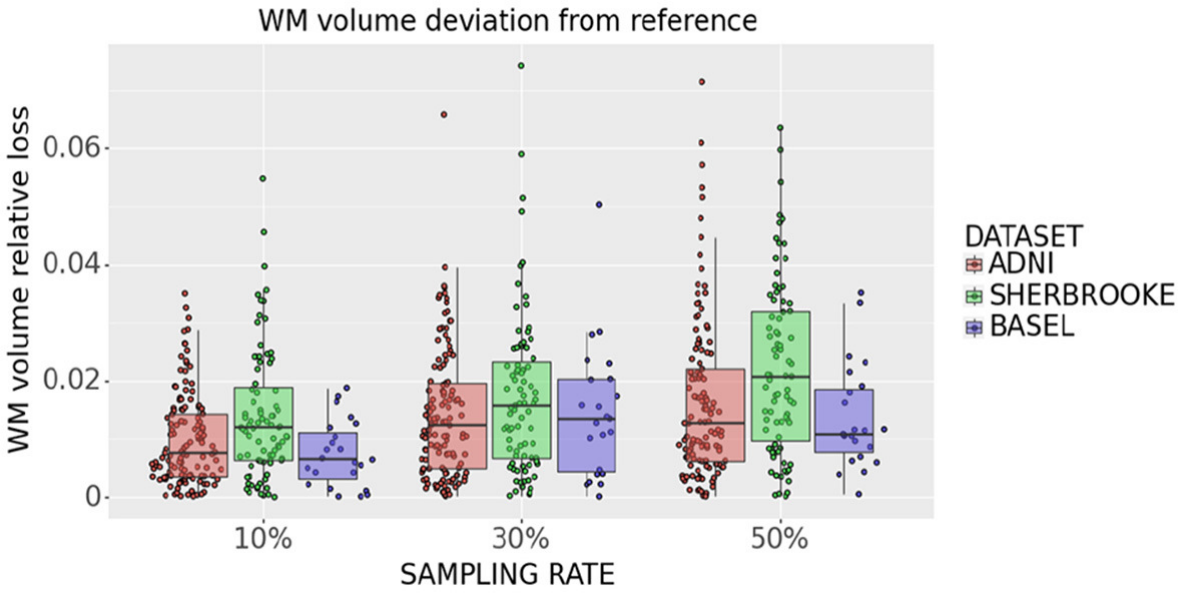
Statistical distribution of WM volume relative loss in all subjects and visits, comparing our three data sets. Colors represent different data sets: ADNI3 (red), Sherbrooke (green), Basel (blue). The *y*-axis refers to the subject distribution of the WM volume relative loss. The *x*-axis refers to the three adopted sampling rates.

White matter analysis in Figure 11 indicates that removing 10% of measures from the reference sequence induces a WM volume loss (median and 95% CI) of (0.76 ± 0.13)% in ADNI3, (0.65 ± 0.17)% in Basel and (1.21 ± 0.73)% in Sherbrooke. Increasing the down-sampling at 30%, the loss in ADNI3 rises to (1.24 ± 0.27)%, in Sherbrooke to (1.61 ± 0.54)%, and in Basel to (1.35 ± 0.68)%. When halving the directions (sampling rate of 50%), the loss stays on (1.27 ± 0.39)% in ADNI3, on (1.09 ± 0.50)% in Basel, and reaches (2.09 ± 0.81)% in Sherbrooke data.

Statistical analysis of the bundle volume across subjects did not show any significant differences.

## 4 Discussion

This study showed that it is possible to significantly reduce dMRI acquisitions with little or no impact on the reliability of the measurements. This could have an important impact on the duration of acquisition protocols, and serve as a source of inspiration to standardize clinical protocols. Our findings were consistent across three data sets with different multi-shell protocols, and corresponded to the acquisition of: six directions at *b* = 300 *s*/*mm*^2^, 22 at *b* = 1,000 *s*/*mm*^2^, and 42 at *b* = 2,000 *s*/*mm*^2^ in the Sherbrooke data set, of four directions at *b* = 500 *s*/*mm*^2^, 34 at *b* = 1,000 *s*/*mm*^2^, and 42 at *b* = 2,000 *s*/*mm*^2^ in ADNI3, and of four directions at *b* = 700 *s*/*mm*^2^, 14 at *b* = 1,000 *s*/*mm*^2^, and 32 at *b* = 2,000 *s*/*mm*^2^ in the Basel data set.

The choice of an optimal number of diffusion-encoding directions and/or acquisition scheme is under debate. Hasan et al. (2001) suggested that more than six directions have no practical advantage, and in Ni et al. (2006), a higher number of diffusion-encoding directions presented no clear advantage to estimate FA and the diffusion tensor. On the other hand, Wang et al. (2011) proved a higher number of directions can improve the intersession reliability, and Jones (2004) demonstrated that 20 and 30 diffusion-encoding directions are needed to estimate FA and the tensor orientation respectively. Furthermore, in Liu et al. (2014) the authors confirmed that increasing the number of directions yields better accuracy and reproducibility. They considered ROIs in the CC and internal capsule and assessed DTI measurements with a fixed number of gradient directions (6, 15, and 32). Tournier et al. (2020) designed an optimal acquisition scheme for neonatal diffusion MRI, maximizing the sensitivity to the information carried by the signal. Gaviraghi et al. (2022) and the majority of the cited works focused on the reconstruction of FA maps on the whole WM, while our work compared two diffusion models (DTI, NODDI) in different WM bundles. To the best of our knowledge, only Lebel et al. (2012) extended such reproducibility study at the level of white matter bundles. The authors reported clear disadvantages in acquiring only six directions, such as a reduced accuracy in the reconstruction of bundle volumes.

However, due to the clear lack of standardized approaches, there is no consensus on the minimum number of directions and their impact on the reproducibility of measurements. It remains unclear whether carefully selecting fewer gradient sampling orientations could provide a better solution than acquiring redundant data. A limited and clear number of gradient directions would be beneficial in terms of shorter acquisition times and address the current lack of a standardized approach to acquisition. In order to address this need, our findings indicated that the acquisition of around 50–80 dMRI volumes (instead of 70–110) was sufficient for an accurate estimate of diffusion metrics' on all WM tracts in two of the three data sets (Basel and Sherbrooke). The accurate estimate of diffusion metrics' profile in ADNI3 was limited to Splenium, RG, OR, ILF, IFO, and Anterior Body. The term dMRI volume refers to any diffusion-weighted image, acquired with a particular b-value and gradient direction.

Of note, in Edde et al. (2023) the authors showed that the variability and reproducibility of measurements based on tractography are bundle-specific. In line with these results, our research suggested that the robustness of fewer sampling orientations could be related to the bundle under scrutiny. For example, looking at the different components of SLF (SLFI or dorsal, SLFII or major, and SLFIII or ventral), multi-shell results indicated that increasing the sampling rate showed a more evident impact on the smallest component (SLFIII). Another example is the CC section, which often presented a distribution with higher variance and higher median values. Along with size, a curved and more complex configuration probably had a certain level of relevance. Moreover, Figures 3–5 showed that some metrics can lose more information and confidence in specific bundles, as was already mentioned for DTI measurements in (Lebel et al., 2012). Consequently, depending on the performance of each bundle, it might be advisable to adjust the sampling protocol to achieve better results.

Considering the average value across a bundle might not affect a metric while the profile could slowly deviate from the reference. Statistical analysis on DTI measures showed that the distribution of bundle profiles in original and down-sampled acquisitions do not show any significant difference. On the other hand, ICvf exhibited other characteristics. The significant *p*-values in the case of three segments of the Posterior Body (and two of ILF) in the Basel data set at 10% sampling could be explained by a deviation in the ICvf profile of a few subjects in that exact part of the bundles (see segments 103–105 in Supplementary Figure S1). The limited sample size could also be a contributing factor (Faber and Fonseca, 2014). The significant divergence in the profiles of ADNI3 subjects (Figure 7) might be related to the fact that the data was acquired across multiple centers (Hatt et al., 2019). Even if ADNI3 protocols were standardized, the different hardware and software of the systems may have had an impact on the sensitivity of the downsampled signal.

Moreover, when applying down-sampling on a data set it is important to investigate any potential loss of WM volume related to the sampling as a potential confounder. Indeed, the loss of WM volume is widely reported as a valuable marker to predict or monitor brain damage (Juhasz et al., 2007; Fletcher et al., 2013; Andravizou et al., 2019; Conrad et al., 2022). The volume loss quantified in our analysis is minimal. Hence, the whole target volume where we estimate different metrics did not change significantly when acquiring a considerably lower number of gradient directions. This was further verified by statistical tests across subjects, which confirmed that the distribution of bundle volumes from down-sampled data was not significantly different to the one derived from the reference data.

Multi-shell metrics seemed to suffer at higher sampling rates. This is visible from both the average values and the profile assessments. Furthermore, we know that DTI needs a consistently smaller number of directions (Lebel et al., 2012). Two findings confirmed this:

1. The average values and profiles analysis indicated a higher similarity to the multi-shell reference.
2. While scores of the protocol with the lowest number of directions (Basel) were worse in NODDI metrics, it seemed to outperform the others in DTI.

Comparable findings were observed in Bland–Altman's agreement analysis. While increasing the sampling rate from 10 to 30% and to 50 led to two and four times the size of LoA (i.e., significant growth in variability) in multi-shell measurements, DTI reported more robust values. On the other hand, the estimated bias was about 2–3 orders of magnitude smaller than the absolute values obtained for all measurements.

Furthermore, we must consider that some of the selected data were collected across multiple centers (Stamoulou et al., 2022). This means that protocols differed not only in terms of the number of directions acquired but also, for example, in terms of the scanner type and sequence parameters. Specifically, the ADNI3 data set (adni.loni.usc.edu) was acquired with three different types of Siemens scanners (3T Prisma, PrismaFit, and Skyra), and subjects present an age distribution which significantly differs from that of Data set 2 and 3. Scanner variability and age (Behler et al., 2021) might have affected the statistical distribution of results and could at least partially explain the higher variance of results within the ADNI3.

It is important to acknowledge some limitations:

1. An intra-session analysis is lacking, so it is not yet clear if dMRI could be used even more as a longitudinal biomarker in neurodegenerative diseases by acquiring significantly fewer directions.
2. The use of only one fiber tracts reconstruction method, since the diffusion sampling scheme has been demonstrated to have an impact on tractography (Schilling et al., 2021).
3. The adoption of one image pre-processing pipeline, among many existing workflows.
4. the lack of gradient non-linearity correction. Taking this effect into account in future studies could strengthen our results, especially in terms of reproducibility (Bammer et al., 2003; Tan et al., 2013).

## 5 Conclusions

We investigated the possibility of saving acquisition time in dMRI protocols by concomitantly reducing the number of acquired directions and estimating reliable metrics. Under the assumption that sub-sampling the number of directions of a given percentage translates into saving the same percentage in acquisition time, our results showed that it is possible to reduce the acquisition time by 30%, with little or no impact on the reliability of the measurements. These findings were consistent across three data sets with different multi-shell protocols, and corresponded to the acquisition of around 50–80 dMRI volumes instead of 70–110.

Down-sampling seemed to impact the white matter tracts differently, so that some bundles, such as SLF, were less affected by down-sampling. The robustness of intra-session down-sampled data should also be assessed in the future, to further warrant the use of dMRI in clinical trials and research activities.

## Data availability statement

The data analyzed in this study is subject to the following licenses/restrictions: ADNI is publicly available. The other two datasets are available upon agreement with the data owner. Requests to access these datasets should be directed at: cristina.granziera@unibas.ch; Maxime.Descoteaux@USherbrooke.ca.

## Ethics statement

The studies involving humans were approved by Sherbrooke dataset: Comité d'éthique de la recherche du CIUSSS de l'Estrie in Sherbrooke, Canada. Basel dataset: Ethikkommission Nordwest- und Zentralschweiz. The studies were conducted in accordance with the local legislation and institutional requirements. The participants provided their written informed consent to participate in this study.

## Author contributions

FS: Investigation, Writing – original draft, Writing – review & editing, Conceptualization, Data curation, Formal analysis, Methodology, Software, Validation, Visualization. SG: Writing – review & editing. EZ: Writing – review & editing. ME: Writing – review & editing, Data curation. MW: Data curation, Writing – review & editing. CG: Writing – review & editing, Data curation. MD: Writing – review & editing, Data curation. MB: Supervision, Writing – review & editing, Conceptualization, Methodology. SM: Conceptualization, Supervision, Writing – review & editing, Methodology.

## Footnotes

^1^Data used in the preparation of this article were obtained from the Alzheimer's Disease Neuroimaging Initiative (ADNI) database (adni.loni.usc.edu). The ADNI was launched in 2003 as a public-private partnership, led by Principal Investigator Michael W. Weiner, MD. The primary goal of ADNI has been to test whether serial magnetic resonance imaging (MRI), positron emission tomography (PET), other biological markers, and clinical and neuropsychological assessment can be combined to measure the progression of mild cognitive impairment (MCI) and early Alzheimer's disease (AD).
