# Supplementary material for: Down-sampling in diffusion MRI: a bundle-specific DTI and NODDI study
Source: Front Neuroimaging. 2024 Mar 28;3:1359589. doi: 10.3389/fnimg.2024.1359589 (PMC11007093; doi:10.3389/fnimg.2024.1359589)
Supplement: Supplementary file 1 [file Data_Sheet_1.PDF]

# Supplementary Material

## 1 SUPPLEMENTARY DATA

### 1.1 Part 1

Figure S1 shows the deviation of the reconstructed profile at segments 103-105 of Posterior Body in Basel data.

The distribution of bundles' average values in ISOvf is shown in the boxplots of Figure S2. ILF and SLFIII were particularly affected by down-sampling: at 10% rate, they presented values between 8% and 18% respectively, while at 50% the mean distribution stays between 20% and 30%. The interquartile ranges seemed to depend on the bundle's choice but reported an increase with a down-sampling rate that is more marked comparing 10% and 50% rates. Down-sampling all protocols discarding 10% of directions induced a mean distribution up to around 18%.

Findings in ISOvf differed from the other metrics because the mean distribution is higher, and the sampling rate seemed to have a greater impact. As a recent work (Tristán-Vega et al., 2022) showed, this could be explained by the fact that compartments related to free water diffusion specifically need directions with b-values lower than 1000. Since those directions are progressively reduced to minimum (3), ISOvf could have lost a relevant amount of information with respect to other metrics.

Figure S3 reports the statistical distributions in RD. Means were below 5%, except for Posterior Body and Splenium, which reached respectively maximum values of around 5.5% and around 7%. SLF presented the lowest means at all sampling rates, with values below 2.5%. Data sampled at 50% from Basel achieved substantially lower average values in most bundles, and their distribution was really close to 10% and 30% sampling. As noticed for MD, CC sections such as Splenium, Isthmus, Anterior and Posterior Body presented a wider interquartile range up to more than 3.5%.

Figure S4 showed the mean distributions in OD. SLF and OR always presented values below 12% at 50% sampling rate and deviated from the reference of about 3-4% at the lowest sampling. The highest means were observed in Splenium, Anterior, and Posterior Body with minimum values around 10% at the lowest sampling rate, going up towards 20% halving the directions. An increase in values when decreasing the number of gradient directions within the same data sets was evident as for the other reported NODDI metrics.

### 1.2 Part 2

Methods agreement in FA in Sherbrooke's subjects of Figure S5 showed that CC presents a bias slightly increasing at 50% rate to -0.002. The limits of agreement followed the same trend growing from around ±0.010 to ±0.012. CST showed a similar behaviour, but limits of agreement were moderately wider at 30% (±0.012).

In the case of MD Figure S6 showed in CC a bias of $\pm 10^{-5}$ when down-sampling over 10% rate. Limits of agreement always stayed around $\pm 3 \cdot 10^{-5}$. In CST the bias increased from $2 \cdot 10^{-6}$ at 10% sampling rate, to about $8 \cdot 10^{-6}$ and $7 \cdot 10^{-6}$ at higher rates. Limits of agreement progressively rose from $22 \cdot 10^{-6}$ to $25 \cdot 10^{-6}$ and $27 \cdot 10^{-6}$ when halving directions.

Figure S7 reports Bland-Altman's plot of ICvf in CC. We observed a bias ranging from 0.002 to 0.004. Limits of agreement consistently widened, increasing the sampling rate from 0.009 at 10% to 0.0012 at 30% and 0.017 at 50%. In CST we noticed a bias of ±0.001 at 10% and 30%, going to 0.003 at 50%. Limits of the agreement were again expanding from a ±0.009 at 10% to around ±0.017-0.018 at higher samplings.

Level of agreement in FA in ADNI3 subjects is shown in Figure S8. CC presented a bias increasing with down-sampling from 0 to -0.003 and -0.005. The limits of agreement were always in the range of ±0.009 and ±0.010. CST presented a higher bias at 30% (-0.004 against -0.001 at other samplings), and limits of agreement grew from ±0.007 at 10% to ±0.010 at higher samplings.

In the case of MD Figure S9 showed in CC we had a bias of $\pm 1 \cdot 10^{-6}$ when down-sampling under 50% and of $2 \cdot 10^{-6}$ at 50%. Limits of agreement stayed around $\pm 27 \cdot 10^{-6}$ up to 30% sampling and rose to $\pm 30 \cdot 10^{-6}$ when halving directions. In CST the bias stayed around 0 and limits of agreement around $\pm 3 \cdot 10^{-6}$ at every sampling rate.

Figure S10 reports models' agreement comparison for ICvf in CC. We observed a bias ranging from ±0.001 up to 30% rate, going to 0.002 at 50%. Limits of agreement consistently widened, increasing the sampling rate from 0.003 at 10% to 0.007 at 30% and 50%. In CST we observed a bias of -0.001 at 10% and -0.002 at 30%, going down to around 0 at 50%. Nonetheless, limits of agreement expanded with sampling rates from ±0.006 to ±0.009 and ±0.012.

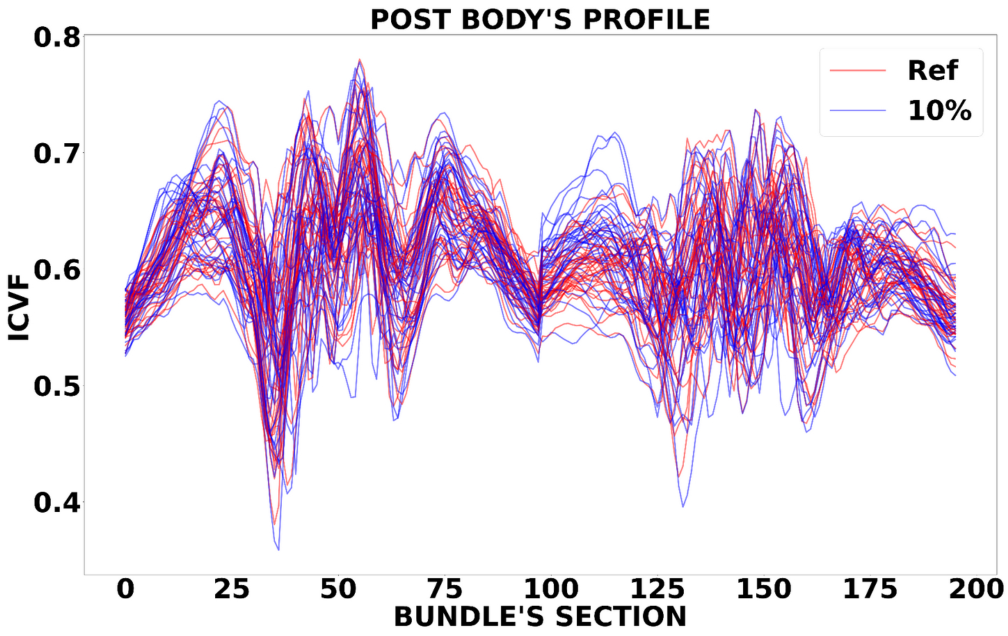


Figure S1: Example of a bundle's ICvf profile reconstruction referring to Posterior in Basel at reference (red) and 10% sampling (blue). The x axis represents the segments of the bundle (196) and the y axis represents the metric.

## REFERENCES

Tristán-Vega, A., París, G., de Luis-García, R., and Aja-Fernández, S. (2022). Accurate free-water estimation in white matter from fast diffusion MRI acquisitions using the spherical means technique. *Magnetic Resonance in Medicine* 87, 1028–1035. doi:10.1002/mrm.28997

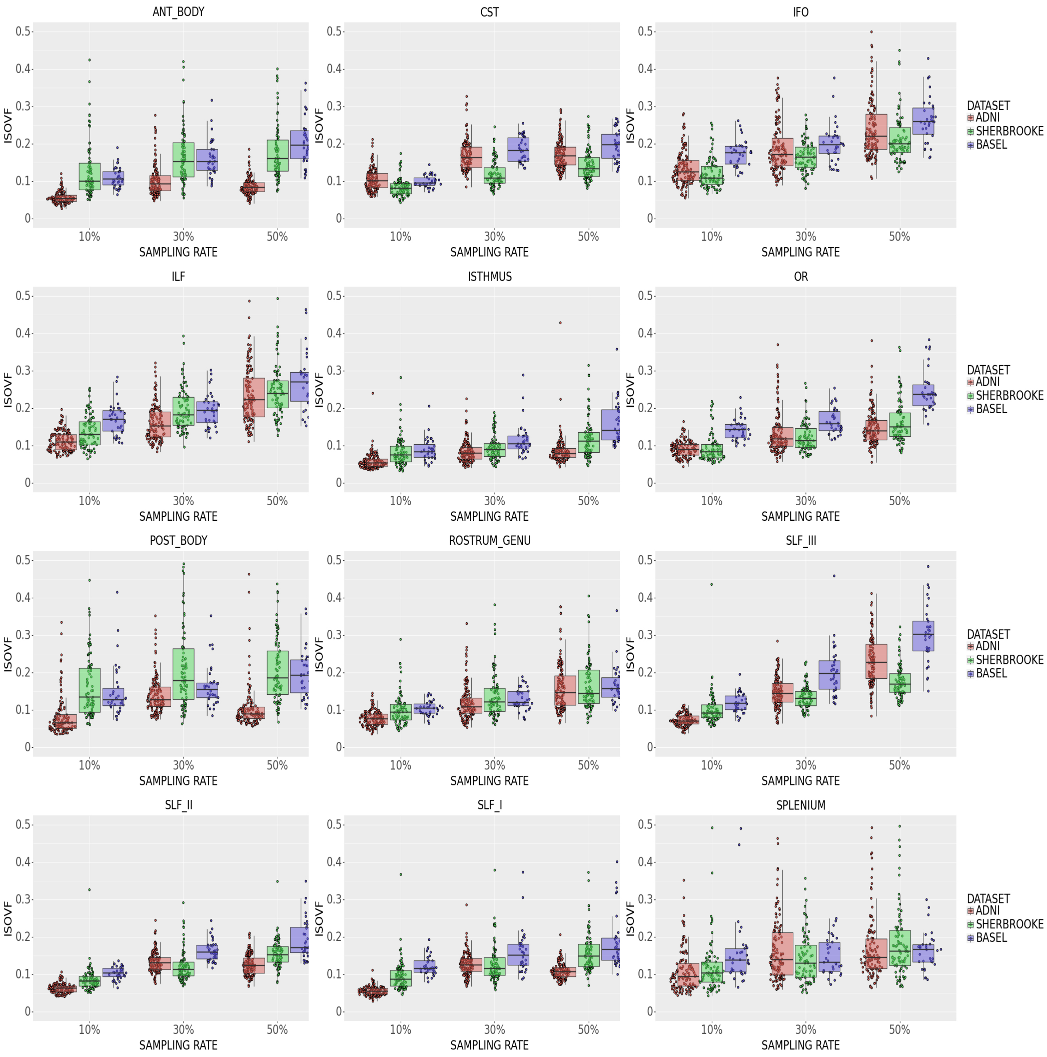


Figure S2: Average values in ISOvf in different bundles comparing our three data sets. Colours of boxes represent different data sets: ADNI3 (red), Sherbrooke (green), Basel (blue). The y axis refers to the subject distribution of the average values for ISOvf in the following bundles: Anterior and Posterior Body, CST, IFO, ILF, Isthmus, OR, Rostrum and Genu, SLF, Splenium. The x axis refers to the three adopted sampling rates.

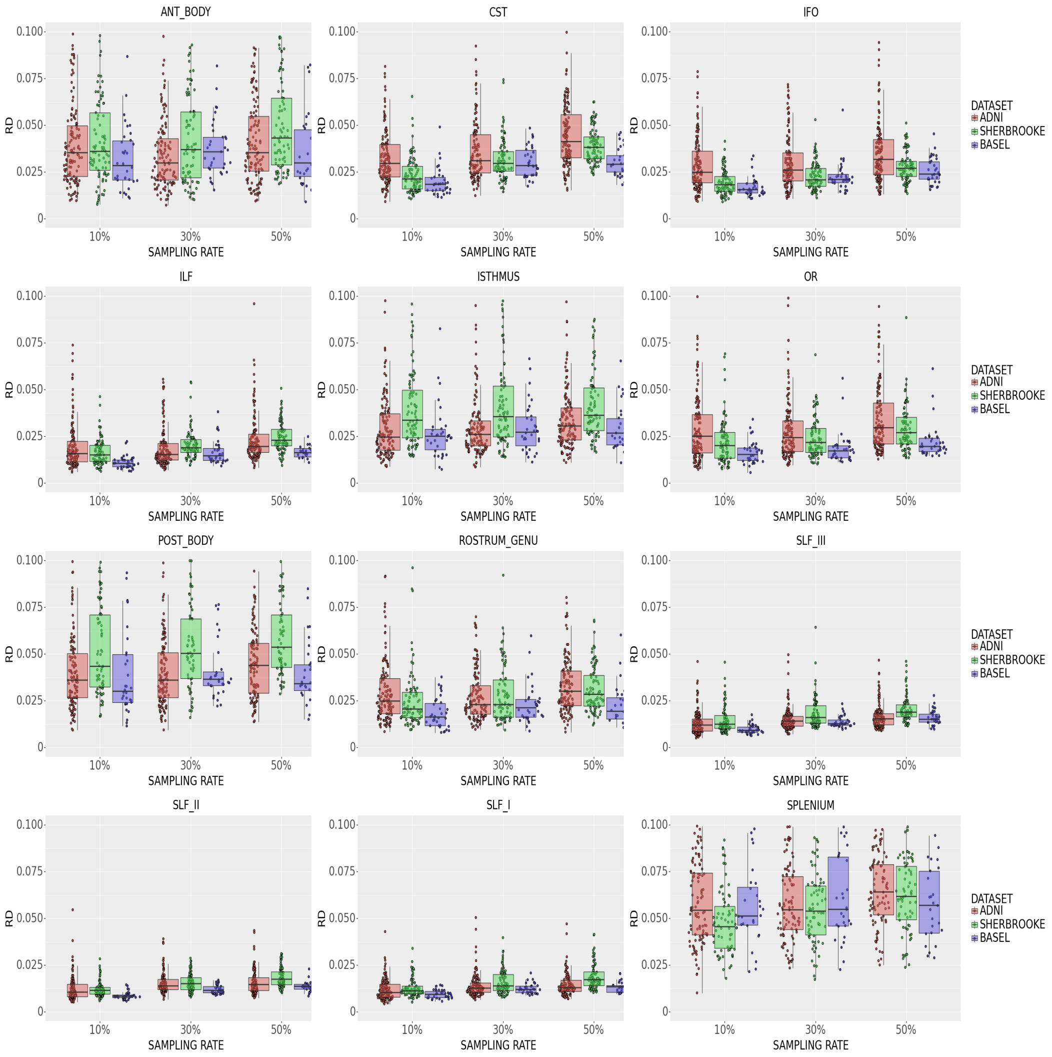


Figure S3: Average values in RD in different bundles comparing our three data sets. Colours of boxes represent different data sets: ADNI3 (red), Sherbrooke (green), Basel (blue). The y axis refers to the subject distribution of the average values for RD in the following bundles: Anterior and Posterior Body, CST, IFO, ILF, Isthmus, OR, Rostrum and Genu, SLF, Splenium. The x axis refers to the three adopted sampling rates.

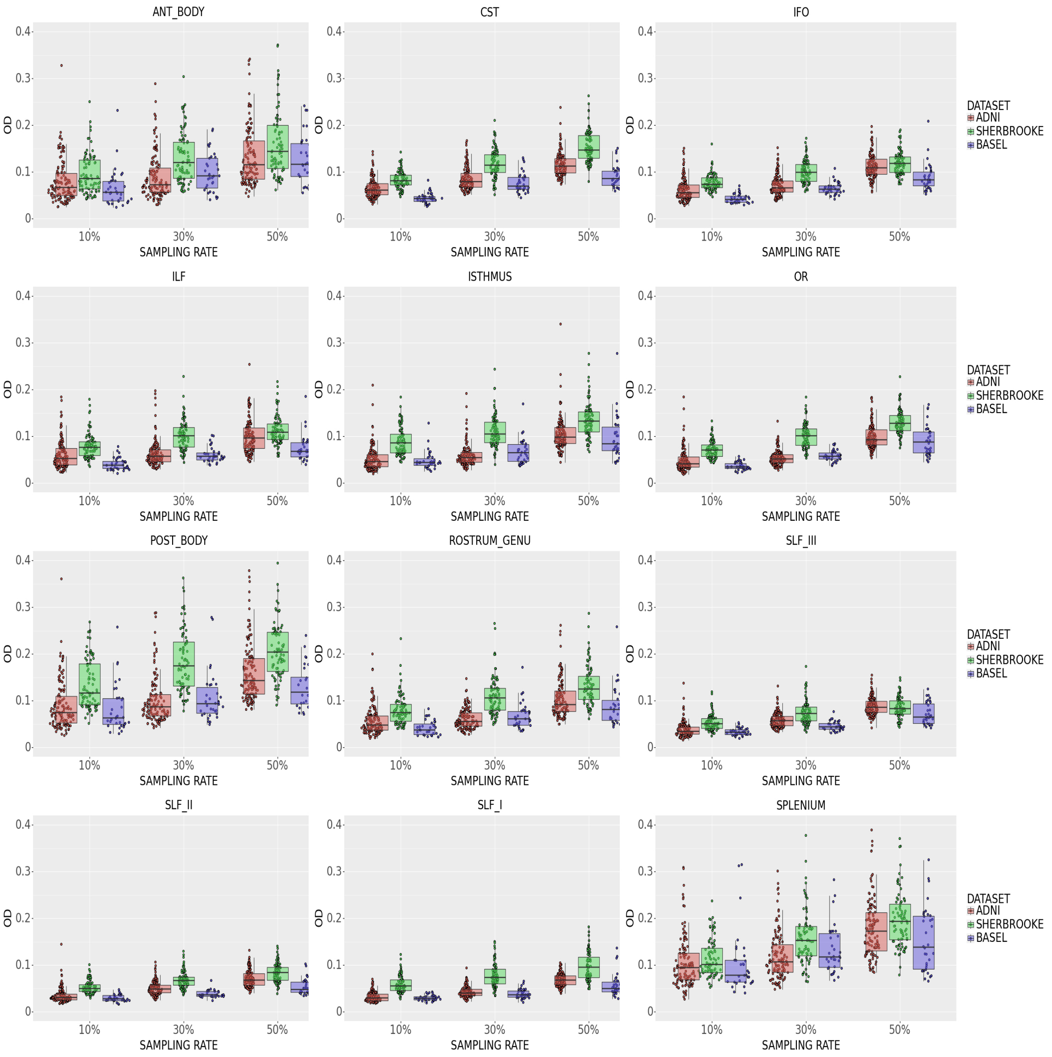


Figure S4: Average values in OD in different bundles comparing our three data sets. Colours of boxes represent different data sets: ADNI3 (red), Sherbrooke (green), Basel (blue). The y axis refers to the subject distribution of the average values for MD in the following bundles: Anterior and Posterior Body, CST, IFO, ILF, Isthmus, OR, Rostrum and Genu, SLF, Splenium. The x axis refers to the three adopted sampling rates.

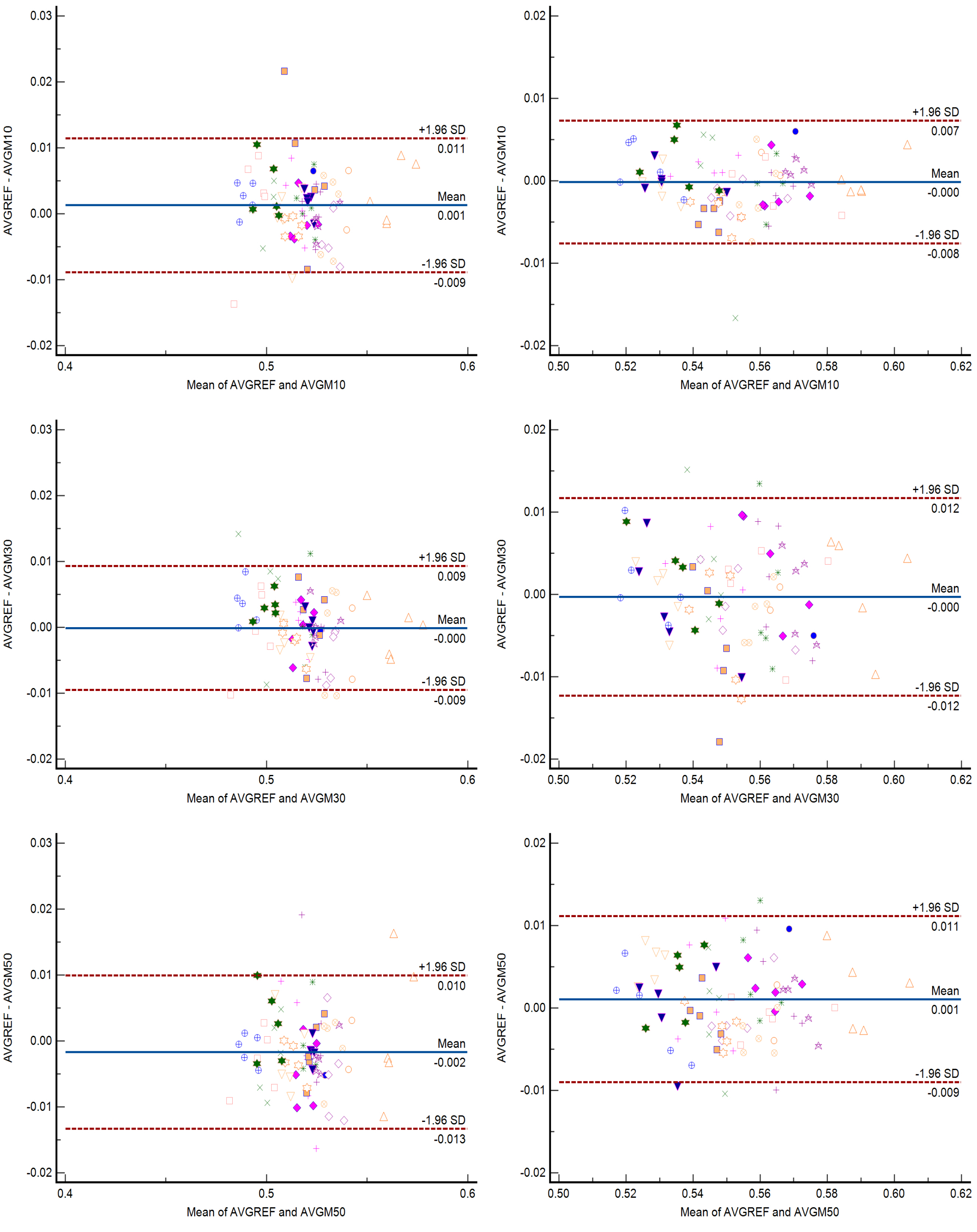


Figure S5: Bland-Altman plots of FA average values in CC (left column) and CST (right column) for Sherbrooke subjects. From top to bottom each plot in the columns compares data obtained from reference sequence (AVGREF) and data down-sampled at 10% (AVGM10), 30% (AVGM30) and 50% (AVGM50). The x axis represents the mean of values measured by the models, the y axis their difference. Visits of a same subject are plotted using the same marker. Blue solid lines correspond to the average difference while red dashed lines are the 95% limits of agreement.

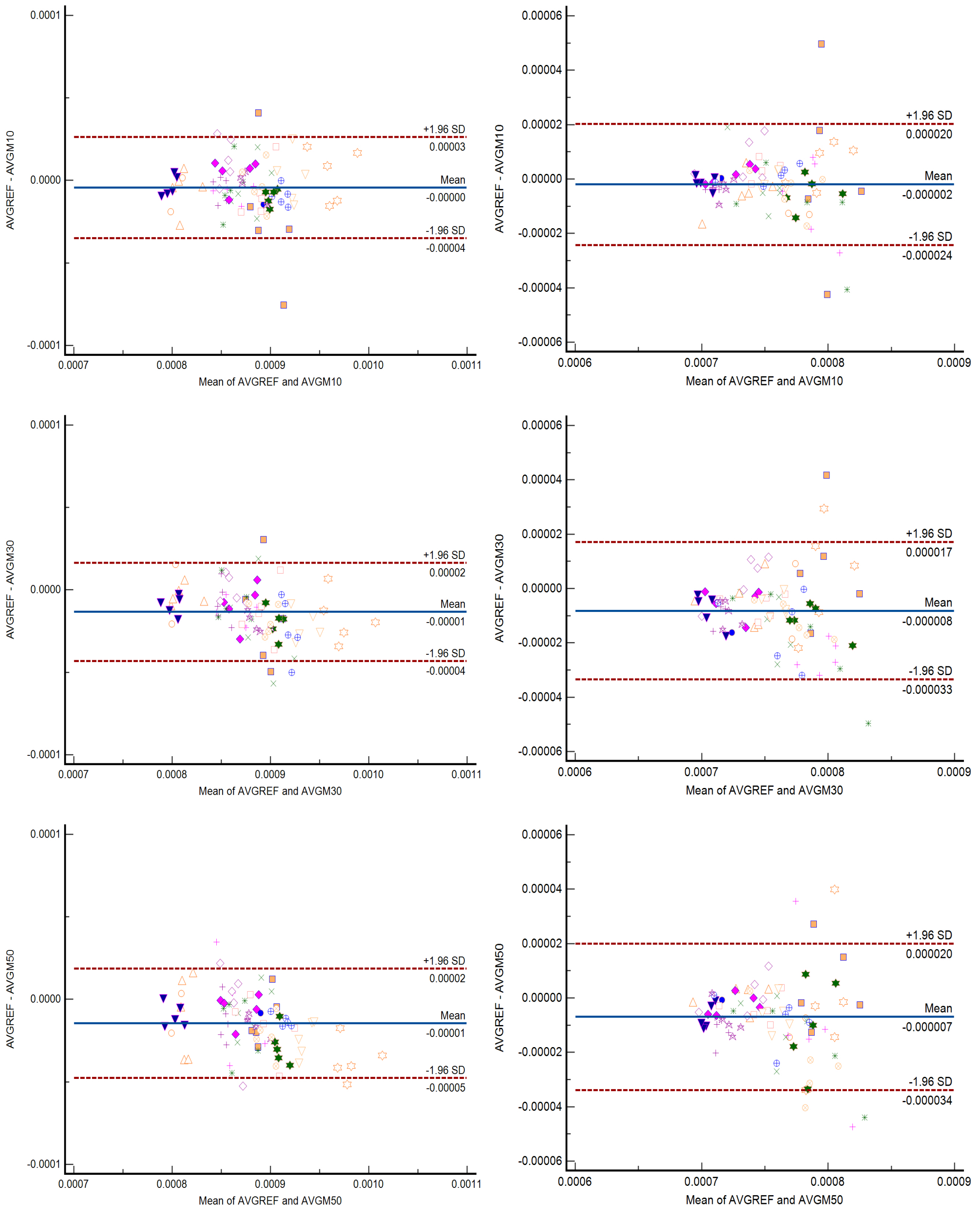


Figure S6: Bland-Altman plots of MD average values in CC (left column) and CST (right column) for Sherbrooke subjects. From top to bottom each plot in the columns compares data obtained from reference sequence (AVGREF) and data down-sampled at 10% (AVGM10), 30% (AVGM30) and 50% (AVGM50). The x axis represents the mean of values measured by the models, the y axis their difference. Visits of a same subject are plotted using the same marker. Blue solid lines correspond to the average difference while red dashed lines are the 95% limits of agreement.

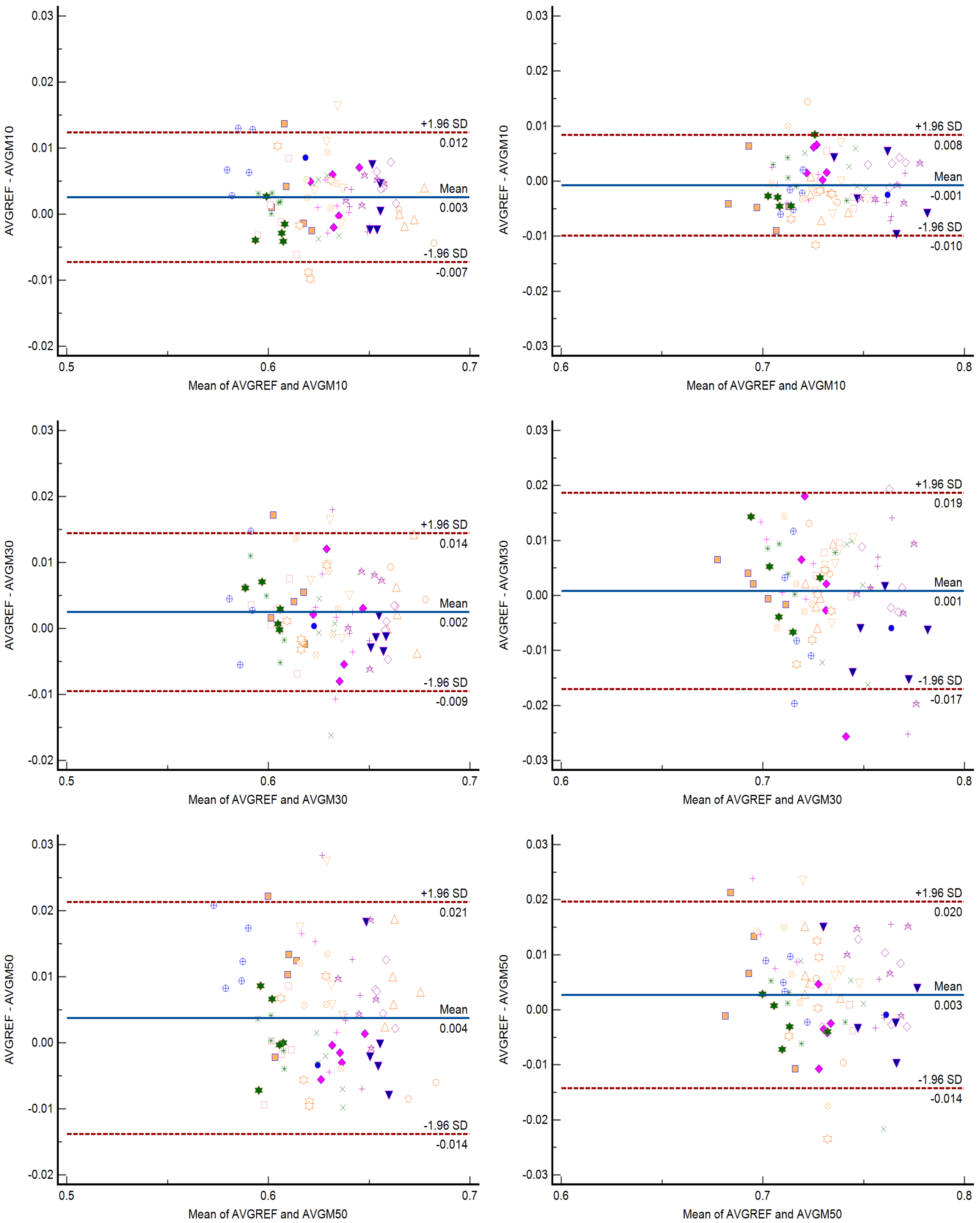


Figure S7: Bland-Altman plots of ICvf average values in CC (left column) and CST (right column) for Sherbrooke subjects. From top to bottom each plot in the columns compares data obtained from reference sequence (AVGREF) and data down-sampled at 10% (AVGM10), 30% (AVGM30) and 50% (AVGM50). The x axis represents the mean of values measured by the models, the y axis their difference. Visits of a same subject are plotted using the same marker. Blue solid lines correspond to the average difference while red dashed lines are the 95% limits of agreement.

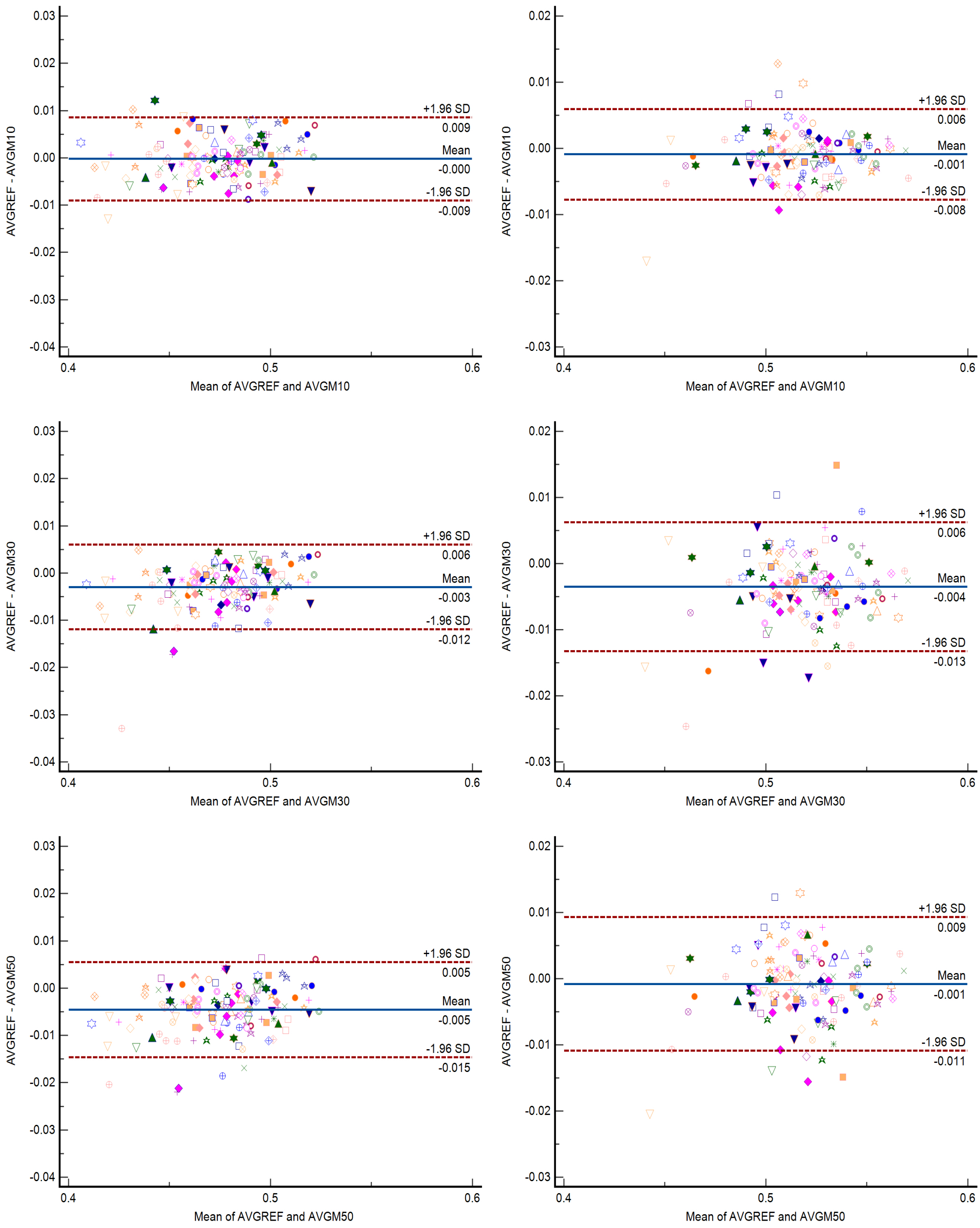


Figure S8: Bland-Altman plots of FA average values in CC (left column) and CST (right column) for ADNI3 subjects. From top to bottom each plot in the columns compares data obtained from reference sequence (AVGREF) and data down-sampled at 10% (AVGM10), 30% (AVGM30) and 50% (AVGM50). The x axis represents the mean of values measured by the models, the y axis their difference. Visits of a same subject are plotted using the same marker. Blue solid lines correspond to the average difference while red dashed lines are the 95% limits of agreement.

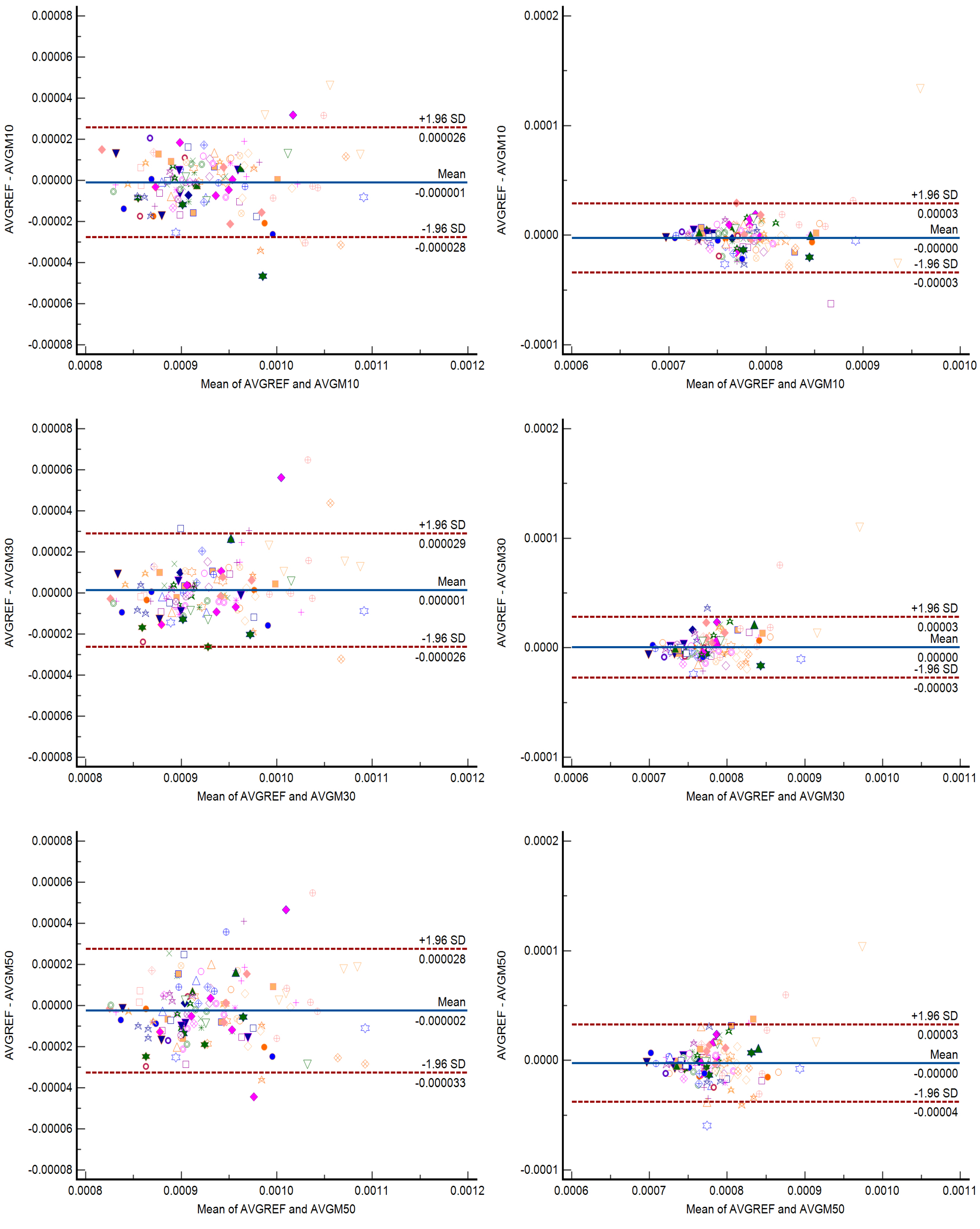


Figure S9: Bland-Altman plots of MD average values in CC (left column) and CST (right column) for ADNI3 subjects. From top to bottom each plot in the columns compares data obtained from reference sequence (AVGREF) and data down-sampled at 10% (AVGM10), 30% (AVGM30) and 50% (AVGM50). The x axis represents the mean of values measured by the models, the y axis their difference. Visits of a same subject are plotted using the same marker. Blue solid lines correspond to the average difference while red dashed lines are the 95% limits of agreement.

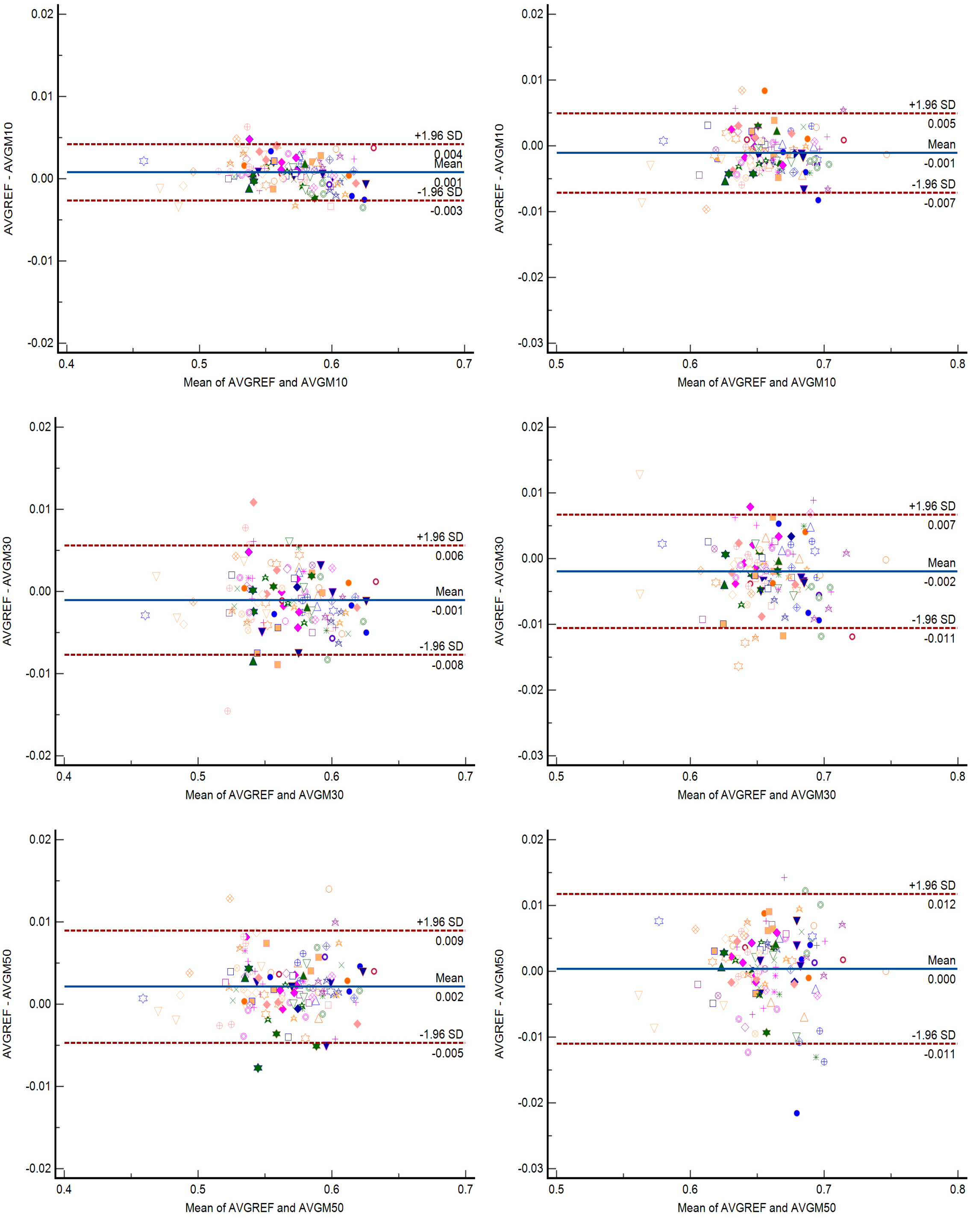


Figure S10: Bland-Altman plots of ICvf average values in CC (left column) and CST (right column) for ADNI3 subjects. From top to bottom each plot in the columns compares data obtained from reference sequence (AVGREF) and data down-sampled at 10% (AVGM10), 30% (AVGM30) and 50% (AVGM50). The x axis represents the mean of values measured by the models, the y axis their difference. Visits of a same subject are plotted using the same marker. Blue solid lines correspond to the average difference while red dashed lines are the 95% limits of agreement.
